# A comprehensive survey of computer vision methods for spatial transcriptomics

**DOI:** 10.1093/bib/bbag255

**Published:** 2026-05-25

**Authors:** Junchao Zhu, Ruining Deng, Junlin Guo, Tianyuan Yao, Siqi Lu, Chongyu Qu, Juming Xiong, Yanfan Zhu, Zhengyi Lu, Yuechen Yang, Marilyn Lionts, Yucheng Tang, Daguang Xu, Yu Wang, Shilin Zhao, Haichun Yang, Yuankai Huo

**Affiliations:** Department of Computer Science, Vanderbilt University, 2301 Vanderbilt Place, Nashville, TN 37235, USA; Weill Cornell Medicine, 1300 York Ave, New York, NY 10065, USA; Department of Electrical and Computer Engineering, Vanderbilt University, 2301 Vanderbilt Place, Nashville, TN 37235, USA; Department of Computer Science, Vanderbilt University, 2301 Vanderbilt Place, Nashville, TN 37235, USA; Department of Computer Science, The College of William and Mary, 200 Stadium Drive, Williamsburg, VA 23185, USA; Department of Electrical and Computer Engineering, Vanderbilt University, 2301 Vanderbilt Place, Nashville, TN 37235, USA; Department of Electrical and Computer Engineering, Vanderbilt University, 2301 Vanderbilt Place, Nashville, TN 37235, USA; Department of Computer Science, Vanderbilt University, 2301 Vanderbilt Place, Nashville, TN 37235, USA; Department of Electrical and Computer Engineering, Vanderbilt University, 2301 Vanderbilt Place, Nashville, TN 37235, USA; Department of Computer Science, Vanderbilt University, 2301 Vanderbilt Place, Nashville, TN 37235, USA; Department of Computer Science, Vanderbilt University, 2301 Vanderbilt Place, Nashville, TN 37235, USA; NVIDIA, 2788 San Tomas Expressway, Santa Clara, CA 95051, USA; NVIDIA, 2788 San Tomas Expressway, Santa Clara, CA 95051, USA; Department of Biostatistics, Vanderbilt University Medical Center, 2525 West End Avenue, Nashville, TN 37203, USA; Department of Biostatistics, Vanderbilt University Medical Center, 2525 West End Avenue, Nashville, TN 37203, USA; Department of Pathology, Microbiology and Immunology, Vanderbilt University Medical Center, 1161 21st Avenue South, Nashville, TN 37232, USA; Department of Computer Science, Vanderbilt University, 2301 Vanderbilt Place, Nashville, TN 37235, USA; Department of Electrical and Computer Engineering, Vanderbilt University, 2301 Vanderbilt Place, Nashville, TN 37235, USA

**Keywords:** computational pathology, spatial transcriptomics, computer vision, medical image analysis

## Abstract

Spatial transcriptomics (ST) enables the simultaneous measurement of gene expression and spatial localization within tissue sections, providing unprecedented opportunities to dissect tissue architecture and functional organization. As a relatively new omics technology, bioinformatics has driven much of the innovation in ST. However, within these frameworks, spatial information is often reduced to locations and relationships between molecular profiles, without fully leveraging the wealth of sub-micron morphological detail and histological knowledge available. Advances in computer vision-based artificial intelligence (AI) are opening exciting new avenues beyond conventional bioinformatics approaches by modeling complex histological patterns and linking morphology to molecular states. More excitingly, they bring fresh perspectives to potentially address key limitations of ST, including its high cost, limited clinical applicability, and reliance on 2D analysis of inherently 3D tissues. For instance, models that predict ST directly from histology images enable virtual sequencing, drastically reducing costs while integrating morphological insights from pathology with molecular biomarkers, thus accelerating clinical translation. Moreover, computer vision techniques can reconstruct pixel-aligned 3D tissue models, overcoming the technical barriers of 2D acquisition and advancing 3D spatial omics analytics. In this paper, we present the first systematic survey of computer vision AI models for ST analytics, categorizing approaches across architectures, learning paradigms, tasks, and datasets, and tracing their technological evolution. We highlight key challenges and future directions, offering a panoramic perspective on vision-driven ST and its potential to transform both basic research and clinical practice. The curated collection of vision-driven ST papers is available at https://github.com/hrlblab/computer_vision_spatial_omics.

## Introduction

 As a rapidly developing multi-omics technology, spatial transcriptomics (ST) enables the simultaneous profiling of gene expression and spatial localization within tissue sections, offering new insights into cellular states, tissue architecture, and functional organization [1, 2]. It has shown broad potential in tumor microenvironment analysis, neuroscience, developmental biology, and clinical pathology [3, 4].

However, as an emerging omics technology, ST still relies heavily on bioinformatics methods for data analysis and model construction. Existing studies mainly focus on expression matrices, spatial adjacency, and gene co-expression networks [5–7]. While effective at the molecular level, these approaches often reduce spatial information to coordinates and graphs, overlooking the fine-grained morphological and histological information embedded in tissue sections [8]. In contrast, histological images contain rich structural cues, from nuclear morphology to tissue organization, that are closely associated with gene expression [9].

Recent advances in computer vision and artificial intelligence (AI) [10, 11] have brought new momentum to ST analysis [12–14]. Deep learning models can capture complex visual patterns and nonlinear relationships between tissue structure and molecular states [8, 15, 16], enabling virtual sequencing and creating new opportunities for molecular research [17, 18]. Beyond 2D analysis, computer vision is also advancing 3D spatial omics [14, 19]. By registering and reconstructing consecutive sections, researchers can build more complete 3D molecular maps of tissues, overcoming the limitations of 2D acquisition [20]. This integration supports more comprehensive studies of tissue development, disease progression, and tumor microenvironments, while laying the foundation for multimodal omics integration.

Despite its great potential, vision-driven ST remains a rapidly evolving field lacking systematic summarization. This paper provides an up-to-date overview of vision-driven ST approaches, focusing on representative models developed between 2020 and 2025. Although early studies demonstrated the feasibility of predicting gene expression from whole slide images (WSIs), most existing reviews focus on ST platforms rather than AI-driven image-based modeling. Therefore, this survey highlights the major methodological advances of vision-driven ST over the past 5 years, summarizes key progress, discusses remaining challenges, and outlines future directions.

**Figure 1:**
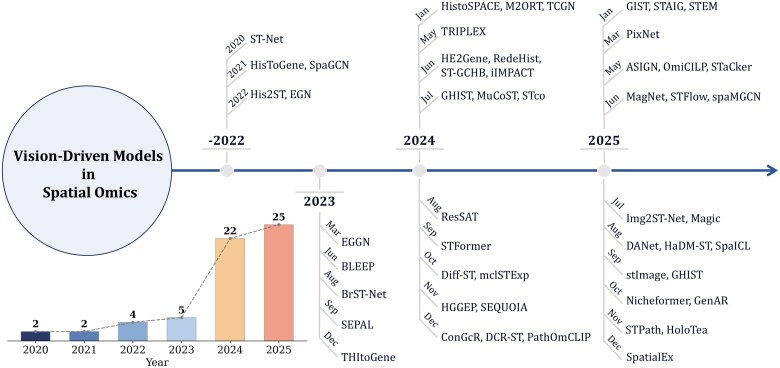
Overview of some well-known vision-driven models for ST from 2020 to 2025.

Figure 1 presents a chronological overview of 46 representative vision-driven ST models published between 2020 and 2025, showing the field’s progression from early exploration to rapid expansion. For clarity, we further categorize these models by targeted spatial resolution and downstream tasks, distinguishing methods for general ST problems from those for specific biological or clinical applications. Notably, inclusion in the timeline reflects existing validation rather than excluding other promising architectures. Meanwhile, advanced paradigms from computer vision and natural language processing are increasingly being adapted to ST research [21–23]. Our contributions include:

1. The first comprehensive review of existing vision-driven ST models, covering methodological background, core architectures, and downstream applications. We summarize recent developments in a hierarchical and structured manner, highlighting the evolution of the field, and the connections across different research directions.
2. Categorization and comparative analysis of models based on real-world application scenarios (Table 1) and their training paradigms (Table 2). We focus on architectural designs, training strategies, data requirements, and task suitability, aiming to provide a clear understanding of each model’s strengths, limitations, and practical scope.
3. Discussion of open challenges and future directions in vision-driven ST. We identify key bottlenecks, emerging trends, and unresolved questions, and offer insights into potential avenues for future research.

## Materials and methods

### Data landscape

#### Spatial transcriptomics data acquisition pipelines

ST maps gene expression to precise locations within intact tissue architecture [24]. Preserving spatial coordinates in tissue sections, it enables *in situ* analysis of cellular states and their interactions with local microenvironments, thereby aligning molecular and morphological information on the same slide [25–28].

**Table 1:** Landscape of vision-driven models and downstream tasks from May 2020 to December 2025, summarizing each model’s backbone architecture, output resolution, and reported tasks, with checkmarks indicating tasks that were quantitatively evaluated.

**Year-Month**	**Architecture**	**Model**	**Resolution**	**GenST**	**Clustering**	**Super-resolution**	**Alignment/3D**
2020-May	GNN	stLearn [12]	Spot-level	–	$\checkmark $	–	–
2020-August	CNN	ST-Net [8]	Spot-level	$\checkmark $	–	–	–
2021-October	GCN	SpaGCN [39]	Spot-level	–	$\checkmark $	–	–
2021-November	Transformer	HisToGene [40]	Spot-level	$\checkmark $	–	$\checkmark $	–
2022-January	MAE+GNN	conST [41]	Spot-level	–	$\checkmark $	–	–
2022-May	CNN	DeepSpaCE [42]	Spot-level	$\checkmark $	–	$\checkmark $	–
2022-July	Transformer+GNN	Hist2ST [18]	Spot-level	$\checkmark $	–	–	–
2022-December	CNN	EGN [15]	Spot-level	$\checkmark $	–	–	–
2023-March	CNN+GNN	EGGN [43]	Spot-level	$\checkmark $	–	–	–
2023-June	CLIP	BLEEP [16]	Spot-level	$\checkmark $	–	–	–
2023-August	CNN+Transformer	BrST-Net [44]	Spot-level	$\checkmark $	–	–	–
2023-September	GNN	SEPAL [45]	Spot-level	$\checkmark $	–	–	–
2023-December	Transformer+GNN	THItoGene [17]	Spot-level	$\checkmark $	–	–	–
2024-January	AE	HistoSPACE [46]	Spot-level	$\checkmark $	–	–	–
2024-January	ViT	M2ORT [47]	Spot-level	$\checkmark $	–	–	–
2024-January	CNN+Transformer+GNN	TCGN [48]	Spot-level	$\checkmark $	–	–	–
2024-March	ViT	TransformerST [49]	Spot-level	–	$\checkmark $	$\checkmark $	–
2024-May	CNN+Transformer	TRIPLEX [50]	Spot-level	$\checkmark $	–	–	–
2024-June	CNN	HE2Gene [51]	Spot-level	$\checkmark $	–	–	–
2024-June	CNN	RedeHist [52]	Cell-level	$\checkmark $	$\checkmark $	–	–
2024-June	CNN+GNN	ST-GCHB [53]	Spot-level	$\checkmark $	–	–	–
2024-June	GLM	iIMPACT [54]	Spot-level	–	$\checkmark $	–	–
2024-July	Transformer	HisToSGE [55]	Spot-level	$\checkmark $	–	$\checkmark $	–
2024-July	GNN	MuCoST [56]	Spot-level	–	$\checkmark $	–	–
2024-July	CLIP	STco [57]	Spot-level	$\checkmark $	–	–	–
2024-August	CNN+Transformer	ResSAT [58]	Spot-level	$\checkmark $	–	–	–
2024-September	Transformer	STFormer [59]	Spot-level	–	$\checkmark $	–	–
2024-October	Diffusion	stDiff [60]	Spot and Cell-level	$\checkmark $	–	–	–
2024-October	CLIP	mclSTExp [61]	Spot-level	$\checkmark $	–	–	–
2024-November	CNN+Transformer	HGGEP [62]	Spot-level	$\checkmark $	–	–	–
2024-November	CNN+Transformer	SEQUOIA [63]	Bulk-level	$\checkmark $	–	–	–
2024-December	CNN+GNN	ConGcR [64]	Spot-level	$\checkmark $	–	–	–
2024-December	CNN+GNN	STMVGAE [65]	Spot-level	–	$\checkmark $	–	–
2024-December	CLIP	PathOmCLIP [66]	Spot-level	$\checkmark $	–	–	–
2025-January	GNN	GIST [67]	Spot and Cell-level	$\checkmark $	$\checkmark $	–	–
2025-January	GNN	STAIG [19]	Spot-level	–	$\checkmark $	–	$\checkmark $
2025-January	Diffusion	STEM [68]	Spot-level	$\checkmark $	–	–	–
2025-March	CNN	PixNet [69]	Spot and Cell-level	$\checkmark $	–	–	–
2025-May	CNN+GNN	ASIGN [14]	Spot-level	$\checkmark $	–	–	$\checkmark $
2025-May	CLIP	OmiCLIP [13]	Spot and Bin-level	$\checkmark $	$\checkmark $	–	$\checkmark $
2025-May	CNN	STaCker [20]	Spot-level	–	–	–	$\checkmark $
2025-June	CNN+GNN	MagNet [70]	Spot and Bin-level	$\checkmark $	–	–	–
2025-June	Flow Matching	STFlow [71]	Spot-level	$\checkmark $	–	–	–
2025-June	AE+GNN	spaMGCN [72]	Spot-level	–	$\checkmark $	–	$\checkmark $
2025-July	CNN	Img2ST-Net [73]	Bin-level	$\checkmark $	–	–	–
2025-July	CLIP	Magic [74]	Spot-level	$\checkmark $	–	–	–
2025-August	CLIP	DANet [75]	Spot-level	$\checkmark $	–	–	–
2025-August	Diffusion	HaDM-ST [76]	Cell-level	$\checkmark $	–	–	–
2025-August	GNN	SpaICL [77]	Spot-level	–	$\checkmark $	–	–
2025-September	CNN	stImage [78]	Spot-level	$\checkmark $	–	–	–
2025-September	CNN	GHIST [79]	Spot and Cell-level	$\checkmark $	$\checkmark $	–	–
2025-October	Transformer	Nicheformer [80]	Spot and Cell-level	$\checkmark $	$\checkmark $	$\checkmark $	$\checkmark $
2025-October	Autoregressive	GenAR [81]	Spot level	$\checkmark $	–	–	–
2025-October	CLIP	UMPIRE [82]	Spot level	$\checkmark $	$\checkmark $	$\checkmark $	$\checkmark $
2025-November	CNN	HiFusion [83]	Spot level	$\checkmark $	–	–	$\checkmark $
2025-November	Transformer	STPath [84]	Spot level	$\checkmark $	$\checkmark $	$\checkmark $	$\checkmark $
2025-November	Flow Matching	HoloTea [85]	Spot level	$\checkmark $			$\checkmark $
2025-December	Transformer	SpatialEx [86]	Spot level	$\checkmark $	$\checkmark $		$\checkmark $
2025-December	CLIP	SCR$^{2}$-ST [87]	Spot level	$\checkmark $			

**Table 2:** Comparison of collected methods for generative spatial omics tasks, summarizing the backbone architecture, output resolution, evaluated datasets, and learning paradigm adopted by each model.

**Model**	**Architecture**	**Resolution**	**Dataset**	**Regression**	**Retrieval**	**Generation**
ST-Net [8]	CNN	Spot-level	Breast-ST [8]	$\checkmark $	–	–
HisToGene [40]	Transformer	Spot-level	HER2 [93]	$\checkmark $	–	–
DeepSpaCE [42]	CNN	Spot-level	–	$\checkmark $	–	–
Hist2ST [18]	Transformer+GNN	Spot-level	HER2 [93], cSCC [92]	$\checkmark $	–	–
EGN [15]	CNN	Spot-level	Breast-ST [8]	$\checkmark $	–	–
EGGN [43]	CNN+GNN	Spot-level	Breast-ST [8]	$\checkmark $	–	–
BLEEP [16]	CLIP	Spot-level	Human-Liver [101]	–	$\checkmark $	–
BrST-Net [44]	CNN+Transformer	Spot-level	Breast-ST [8]	$\checkmark $	–	–
SEPAL [45]	GNN	Spot-level	Breast-ST [8]	$\checkmark $	–	–
THItoGene [17]	Transformer+GNN	Spot-level	HER2 [93], cSCC [92]	$\checkmark $	–	–
HistoSPACE [46]	AE	Spot-level	HER2 [93], Breast-ST [8]	$\checkmark $	–	–
M2ORT [47]	ViT	Spot-level	HER2 [93], cSCC [92], Breast-ST [8]	$\checkmark $	–	–
TCGN [48]	CNN+Transformer+GNN	Spot-level	HER2 [93]	$\checkmark $	–	–
TRIPLEX [50]	CNN+Transformer	Spot-level	Breast-ST [8], HER2 [93], cSCC [92], BC1 [102], BC2 [102]	$\checkmark $	–	–
HE2Gene [51]	CNN	Spot-level	Breast-ST [8], HER2 [93]	$\checkmark $	–	–
RedeHist [52]	CNN	Cell-level	Breast-Xenium [102]	$\checkmark $	–	–
ST-GCHB [53]	CNN+GNN	Spot-level	DLPFC [95]	$\checkmark $	–	–
HisToSGE [55]	Transformer	Spot-level	DLPFC [95], BC1 [102], BC2 [102], MOB [30]	$\checkmark $	–	–
GHIST [79]	CNN	Cell-level	Breast-Xenium [102]	$\checkmark $	–	–
STco [57]	CLIP	Spot-level	HER2 [93], cSCC [92]	–	$\checkmark $	–
ResSAT [58]	CNN+Transformer	Spot-level	MOB [30]	$\checkmark $	–	–
stDiff [60]	Diffusion	Spot and Cell-level	Breast-Xenium [102], Breast-ST [8]	–	–	$\checkmark $
mclSTExp [61]	CLIP	Spot-level	HER2 [93], cSCC [92]	–	$\checkmark $	–
HGGEP [62]	CNN+Transformer	Spot-level	HER2 [93], cSCC [92]	$\checkmark $	–	–
PathOmCLIP [66]	CLIP	Spot and Cell-level	HEST-1K [99]	$\checkmark $	–	–
STEM [68]	Diffusion	Spot-level	HER2 [93], Human-Kidney [94]	–	–	$\checkmark $
PixNet [69]	CNN	Spot and Cell-level	Breast-ST [8], HER2 [93], Breast-HD [96]	–	–	$\checkmark $
ASIGN [14]	CNN+GNN	Spot-level	Breast-ST [8], HER2 [93], DLPFC [95]	$\checkmark $	$\checkmark $	–
OmiCLIP [13]	CLIP	Spot and Bin	OmiCLIP-ST [13]	–	$\checkmark $	–
MagNet [70]	CNN+GNN	Spot and Bin	CRC-HD [97], VUMC-Kidney-HD [70]	$\checkmark $	–	–
STFlow [71]	Flow Matching	Spot-level	HEST-1K [99], STImage-1K4M [100]	–	–	$\checkmark $
Img2ST-Net [73]	CNN	Bin	CRC-HD [97], Breast-HD [96]	–	–	$\checkmark $
Magic [74]	CLIP	Spot-level	SpatialTME [103]	$\checkmark $	–	–
DANet [75]	CLIP	Spot-level	HER2 [93], Human-Liver [101]	–	$\checkmark $	–
HaDM-ST [76]	Diffusion	Cell-level	Breast-Xenium [102], Mouse-Brain-Xenium [101]	–	–	$\checkmark $

**Figure 2:**
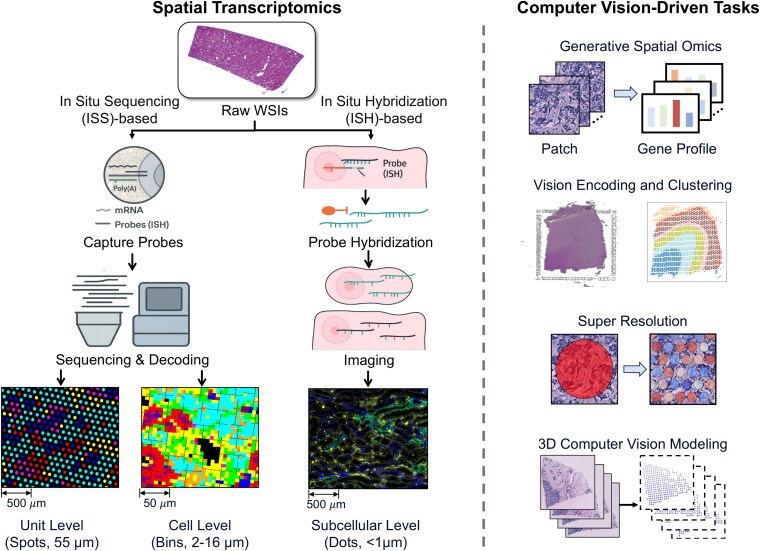
ST data acquisition and computer vision methods. Left: Two acquisition families. (a) *In situ* sequencing (ISS)-based capture producing 55 $\mu $m spot-level maps and high-resolution binned maps (2/8/16 $\mu $m). (b) *In-situ* hybridization yielding subcellular readouts (<1 $\mu $m). Right: Representative downstream tasks enabled by histology and ST, including (1) Generative Spatial Omics, (2) Vision Feature Encoding and Clustering, (3) Super Resolution, (4) 3D Computer Vision Modeling.

As shown in Fig. 2, ST technologies range from lower-resolution platforms such as Visium ( 55 $\mu $m spot size) to high-resolution and subcellular-scale platforms such as Xenium and CosMx. They can be broadly divided into *in situ* sequencing (ISS)-based and *in situ* hybridization (ISH)-based methods [29–33]. ISS provides broad tissue coverage through spatially barcoded mRNA capture and sequencing but is limited by spot size, which may mix signals from multiple cells. ISH achieves single-cell or subcellular resolution through fluorescent probes and iterative imaging.

ST has been widely applied in tumor microenvironment analysis, neural development, immune profiling, pathology, and integrative biomedical research [34–36]. It also supports studies of tissue zonation and cell–cell spatial interactions [37], and is increasingly integrated with single-cell RNA sequencing and digital pathology imaging to improve biological interpretability and predictive power [8, 38].

Despite rapid progress, ST analysis still faces major challenges, including sparse high-dimensional expression matrices, cross-platform batch effects, spatial misalignment, and limited standardized annotations [1, 24, 88]. To address these issues, a variety of computational strategies have been proposed [89–91].

#### Evolution of public spatial transcriptomics datasets

Over the past decade, public ST datasets have evolved rapidly, with major improvements in resolution, scale, and annotation, as summarized in Table 4. Early ST datasets were mainly generated at the spot level, where each capture area aggregates signals from multiple cells [30, 92–94]. Representative examples include mouse olfactory bulb [30], cSCC [92], and DLPFC [95]. These early resources usually contained limited samples and mainly supported tasks such as gene expression prediction. With the emergence of high-resolution platforms, ST has gradually advanced toward bin-level and single-cell–level data. For instance, Breast-HD [96] and CRC-HD [97] provide bin-level data, while Breast-Xenium and Mouse-Brain-Xenium [98] achieve single-cell resolution, enabling finer characterization of cell–cell interactions.

**Table 4:** Summary of representative ST datasets used in image-to-ST studies, reporting the release date, dataset name, profiling resolution, primary organs, numbers of samples, slides and spots, and typical applications for each dataset.

**Year-Month**	**Dataset**	**Title**	**Resolution**	**Organ**	**Sample**	**Slides**	**Spot**	**Application**
2016-July	MOB [30]	Visualization and analysis of gene expression in tissue sections by ST	Spot-level	Brain	1	12	4,147	ST Prediction
2020-July	cSCC [92]	Multimodal analysis of composition and spatial architecture in human squamous cell carcinoma	Spot-level	Skin	4	12	8,671	Alignment/3D, ST Prediction
2020-August	Breast-ST [8]	Integrating spatial gene expression and breast tumor morphology via deep learning	Spot-level	Breast	23	68	30,655	Alignment/3D, ST Prediction
2021-February	DLPFC [95]	Transcriptome-scale spatial gene expression in the human dorsolateral prefrontal cortex	Spot-level	Brain	3	12	47,681	Clustering, Alignment/3D, ST Prediction
2021-October	HER2 [93]	Spatial deconvolution of HER2-positive breast cancer delineates tumor-associated cell type interactions	Spot-level	Breast	8	36	13,620	Alignment/3D, ST Prediction
2023-July	Human-Kidney [94]	An atlas of healthy and injured cell states and niches in the human kidney	Spot-level	Kidney	24	24	74,220	ST Prediction
2023-July	Human-CCS [125]	Spatially resolved multiomics of human cardiac niches	Spot-level	Heart	25	41	100,539	ST Prediction, Clustering
2023-November	Breast-HD [96]	Visium HD enables spatial discovery in FFPE human breast cancer at single-cell scale	Bin-level	Breast	4	4	-	ST Prediction
2023-December	BC1 [102], BC2 [102]	High-resolution mapping of the tumor microenvironment using integrated single-cell, spatial and in situ analysis	Spot-level	Breast	2	2	6,331	ST Prediction
2023-December	Breast-Xenium [102]	High-resolution mapping of the tumor microenvironment using integrated single-cell, spatial and in situ analysis	Cell-level	Breast	3	3	-	ST Prediction
2023-December	CRC-ST [126]	Molecular cartography uncovers evolutionary and microenvironmental dynamics in sporadic colorectal tumors	Spot-level	Bowel	31	41	117,216	ST Prediction, Clustering
2024-April	SpatialTME [103]	The Web-Based Portal SpatialTME Integrates Histological Images with Single-Cell and Spatial Transcriptomics to Explore the Tumor Microenvironment	Spot-level	Multi-organ (12)	26 cohort	296	-	Multi-tasks
2024-June	HEST-1k [99]	HEST-1k: A Dataset for Spatial Transcriptomics and Histology Image Analysis	Multi-level	Multi-organ (26)	153 cohort	1,229	2.1M	Multi-tasks
2024-June	STImage-1K4M [100]	STimage-1K4M: A histopathology image-gene expression dataset for ST	Multi-level	Multi-tissue (50)	121 cohort	1,149	4.2M	Multi-tasks
2025-March	Mouse-Brain-Xenium [98]	Optimizing Xenium *In Situ* data utility by quality assessment and best-practice analysis workflows	Cell-level	Brain	1	4	-	ST Prediction
2025-May	OmiCLIP-ST [13]	A visual–omics FM to bridge histopathology with ST	Multi-level	Multi-organ (32)	113 cohort	-	2.2M	Multi-tasks
2025-June	CRC-HD [97]	High-definition spatial transcriptomic profiling of immune cell populations in colorectal cancer	Bin-level	Bowel	5	5	-	ST Prediction

At the same time, dataset scale has expanded substantially. Early studies typically contained only tens of thousands of spots, whereas recent resources include millions of spots across more than a hundred cohorts. Multi-level datasets such as HEST-1k [99], STImage-1K4M [100] further integrate multiple resolutions within unified frameworks. For example, HEST-1k contains 153 cohorts with about 2.1 million spots, while STImage-1K4M covers 121 cohorts and $\sim $4.2 million spots. This expansion provides a stronger basis for robust analysis and large-model training. Supported by these enriched datasets, ST tasks have also become increasingly diverse. What initially focused on gene expression prediction has expanded to include 3D registration and multi-task benchmarking.

### Vision-driven tasks for spatial transcriptomics

Advances in AI and computer vision have provided powerful tools for medical image analysis [104–107]. Recently, vision-driven ST models have emerged as a promising approach to overcome experimental limitations and expand data accessibility [8, 14, 16], driving progress in a range of downstream ST tasks [15, 39, 40, 42]. In this section, we review representative vision-driven tasks in ST and summarize how image-derived features are used to infer molecular landscapes.

#### Generative spatial omics

Generative spatial omics forms the foundation of this field by exploiting morphology–molecular correlations to infer gene expression directly from histology images. Early models such as ST-Net [8] and HisToGene [40] mainly used transfer learning for gene expression prediction, while later methods introduced contrastive and multimodal learning to better align visual and omics representations [16, 61]. With larger datasets and stronger models, recent approaches have further advanced this paradigm. For example, OmiCLIP [13] combines large-scale contrastive learning and multimodal pretraining to build a more general and robust translation framework. Generative spatial omics supports downstream analysis and serves as a key bridge between imaging and omics. Representative paradigms and frameworks are reviewed in Section “Learning manners for generative spatial omics”.

#### Vision feature encoding and clustering

Vision feature encoding is a fundamental step in vision-driven ST, transforming histological textures, and tissue architecture into informative embeddings. Based on these embeddings, clustering becomes a key downstream task for revealing cellular and molecular heterogeneity in space. By improving the alignment between histological morphology and molecular expression, image-derived features help uncover important biological patterns. Representative methods such as SpaGCN [39], SEPAL [45], and STAIG [19] integrate visual features with graph-based modeling to better characterize local tissue structures and identify spatial domains. These studies highlight the central role of visual feature encoding in linking histological organization with molecular expression and establish clustering as a core task for spatial pattern discovery and tissue microarchitecture analysis [49, 52, 77].

#### Super resolution

Early ST platforms were limited by low spot resolution. To address this issue, super-resolution methods have been developed to computationally refine spatial maps with the help of histological structure cues. Models such as HiST2ST [18] and DeepSpaCE [42] can infer gene expression at sub-spot or even single-cell resolution, improving the continuity and accuracy of molecular landscapes. Meanwhile, benchmarking is also moving toward high-resolution settings. Methods such as MagNet [70] and PixNet [69] extend training and evaluation protocols for high-resolution ST data, further enhancing model generalization and robustness.

#### 3D computer vision modeling

To better capture the full structural complexity of biological tissues, recent studies have introduced 3D modeling and reconstruction frameworks that integrate serial histological sections into volumetric molecular atlases [108]. By modeling inter-slice spatial continuity and morphological consistency, these methods move beyond the limitations of 2D analysis. Representative frameworks such as HisToSPACE [46] and STaCker [20] reconstruct organ-level spatial context and continuous molecular landscapes by aligning image and expression features across adjacent slices. Furthermore, ASIGN [14] incorporates 3D spatial relationships and shifts the task from conventional 2D WSI-ST prediction to partially observed 3D volumetric WSI-ST imputation.

#### Key objectives for vision-omics methods

We organize the reviewed studies in Table 3 according to their primary research objectives, with summarized categories into three broader levels, including inference and spatial characterization, biological interpretation and clinical translation, and emerging lifecycle-spanning paradigms.

**Table 3:** Taxonomy of major computational objectives in ST research, organizing representative methods by their primary research objectives together with the emergence year of each direction and the corresponding representative studies.

**Primary Objective**	**Since**	**Methods**
Morphology–Transcriptome Mapping	2020	ST-Net [8], HisToGene [40], DeepSpaCE [42], EGN [15], EGGN [43], BLEEP [16], BrST-Net [44], SEPAL [45], THItoGene [17], HistoSPACE [46], M2ORT [47], TCGN [48], TRIPLEX [50], ST-GCHB [53], STco [57], ResSAT [58], stDiff [60], HGGEP [62], STEM [68], PixNet [69], ASIGN [14], STaCker [20], MagNet [70], STFlow [71], Magic [74], HaDM-ST [76], GenAR [81], HiFusion [83]
Spatial Heterogeneity and TME Characterization	2020	stLearn [12], TransformerST [49], HE2Gene [51], RedeHist [52], HisToSGE [55], MuCoST [56], mclSTExp [61], ConGcR [64], STMVGAE [65], STAIG [19], spaMGCN [72], SpaICL [77], stImage [78]
Gene Set and Pathway Association/SVG Detection	2021	SpaGCN [39], conST [41], Hist2ST [18], iIMPACT [54], GIST [67]
Prognostic and Diagnostic Feature Discovery	2024	SEQUOIA [63], PathOmCLIP [66], DANet [75], GHIST [79]
Foundation Models	2024	STFormer [59], OmiCLIP [13], scGPT-spatial [109], HEIST [110], SToFM [111], SpaFoundation [112], FmH2ST [113], Nicheformer [80], STPath [84], spEMO [114], UMPIRE [82], SpatialEx [86], SEAL [115]
Experimental Design Optimization	2024	SOFisher [116], S2-omics [117], SCR$^{2}$-ST [87]
Automated Analysis Workflow	2024	QuST-LLM [118], CellAgent [119], STAgent [120], ChatSpatial [121], EnsAgent [122], stMCP [123]

##### Morphological inference and spatial characterization

The first two categories address a core question that how to connect tissue morphology with spatial molecular information and to understand tissue spatial organization. Since 2020, both directions have remained highly active and laid the foundation for subsequent developments. Among them, morphology–transcriptome mapping focuses on inferring spatial gene expression directly from H&E histology images. Researches in this direction have progressed from early feasibility demonstrations [8] to stronger modeling of spatial dependencies [40], cross-modal alignment [16], and generative modeling [68]. In contrast, spatial heterogeneity and microenvironment characterization focus on how tissues are spatially organized. Methods in this category [12, 19] are designed for tasks such as spatial domain identification and tumor microenvironment characterization, extending the field from molecular prediction to tissue-level spatial understanding, supporting analysis of cellular ecosystems.

##### Biological interpretation and clinical translation

Building on foundational inference and spatial characterization, the next two objective categories further push the field from molecular prediction toward biological interpretation and clinical application. Among them, Gene Set and Pathway Association/Spatially Variable Gene Detection focuses on biologically interpretable problems, such as identifying spatially variable genes [39], characterizing spatial pathway activity [41], and revealing gene–pathway associations. Compared with direct expression prediction, this category places greater emphasis on biological knowledge and is better suited for mechanistic discovery and functional interpretation.

Furthermore, prognostic and diagnostic feature discovery reflects the field’s growing shift toward clinical translation. This category focuses on linking spatial molecular features to patient prognosis [63] and diagnostic tasks [66, 79], enabling vision-omics methods to move beyond molecular estimation toward clinically meaningful applications. Unlike the previous category, which emphasizes biological interpretation, this direction gives greater weight to translational relevance and practical utility, highlighting a broader shift from predicting molecular signals to using them for clinical decision-making [124].

##### Lifecycle-spanning paradigms and temporal trends

Beyond categories centered on specific scientific questions, the past 2 years have also seen three directions with broader methodological significance: reinforcement learning (RL)-based experimental design optimization, automated analysis workflows, and foundation models (FM). Unlike task-specific methods, these directions address different stages of the ST research pipeline, pushing vision-omics from isolated prediction tasks toward a more general paradigm.

Experimental design optimization and automated analysis workflows extend the field toward the upstream and downstream stages of research, respectively. The former [116, 117] focuses on where to measure and what to prioritize under constraints such as budget, cost, and feasibility, improving the efficiency and information yield of spatial omics experiments. The latter [119, 123] explores how large language models and agent-based systems can organize complex multi-step pipelines, making ST analysis more automated, modular, and interactive. Together, these directions show that vision-omics is expanding from model outputs to the broader chain of data acquisition, analytical execution, and result interpretation.

Foundation models, by contrast, are not designed for a single downstream task but aim to learn general-purpose transferable across applications. Whether for expression prediction, spatial clustering, cross-modal retrieval, or clinical inference, methods in this category [13, 110, 111] all seek stronger generalization through large-scale pretraining. Their rise reflects a broader shift from dataset-specific and task-specific supervised learning toward more general representation learning.

This taxonomy also reveals a clear stage-wise pattern. Morphology-transcriptome mapping and spatial heterogeneity characterization emerged earliest, whereas most other categories developed mainly after 2024. This shift suggests that the field is moving beyond its early prediction-centered framework toward a more comprehensive methodological landscape spanning biological interpretation, clinical translation, experimental decision-making, and workflow automation, marking a more mature stage of vision-omics ST research.

### Learning manners for generative spatial omics

**Figure 3:**
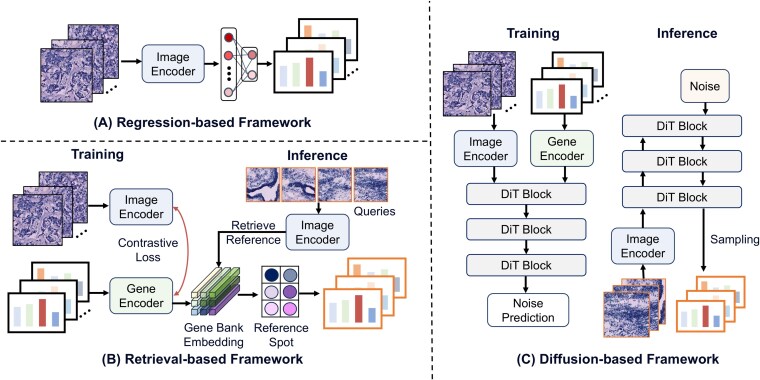
Learning paradigms for generative spatial omics task. Current learning paradigms mainly include three forms: (A) Regression-based prediction which directly maps histology tiles to gene expression profiles; (B) Retrieval-based searching that aligns image–gene representations and retrieves nearest reference spots to form predictions; (C) Diffusion-based generation which performs conditional noise prediction with a DiT and, at inference, denoises from noise to generate expression maps.

Generative spatial omics is one of the most widely studied tasks in vision-driven models. Existing approaches can be broadly grouped into three types: (i) regression-based methods, which directly learn the mapping from image features to gene expression; (2) retrieval-based contrastive learning methods, which align image and expression spaces and predict expression from large-scale databases; and (3) diffusion-based generative frameworks, which reconstruct high-dimensional expression distributions during inference. Figure 3 summarizes the typical training and inference workflows of these three strategies. In this section, we review these mainstream paradigms and discuss representative methods that extend them.

#### Regression-based modeling

Regression-based approaches are among the earliest and most widely used strategies for Image-to-ST translation. They formulate gene expression prediction as a multi-dimensional regression task, where features extracted from histology images are mapped into the gene expression space through regression layers. An early representative work, ST-Net [8], first introduced this idea. ST-Net employs a pretrained CNN backbone [127] to extract image features, which are then projected into the gene expression space through linear layers. Formally, given a histology image $I$ with feature $f(I)$, the predicted gene expression vector $\hat{y}$ can be expressed as: 


(1) \begin{align*}& \hat{y} = W \cdot \mathrm{MLP}(f(I)) + b,\end{align*}

where $W$ and $b$ denote weights and bias of regression layer.

However, modeling each spot independently limits the capture of spatial dependencies, and performance is often constrained by feature quality. To address these issues, later studies introduced strategies to enhance spatial association modeling [14, 18, 40, 128] and feature representation [15, 43, 48]. HisToGene [40] and His2ST [18] incorporate spatial coordinates to connect spots and refine spatial representations. EGN [15] and EGGN [43] further construct connections based on image similarity, bringing visually similar spots closer in feature space to improve prediction. ASIGN [14] extends this idea into 3D space by jointly modeling spatial relationships and feature similarity.

#### Retrieval-based alignment

The success of Contrastive Language-Image Pretraining (CLIP) in vision-language modeling [129] has opened new possibilities for joint image-ST representation learning. ST can be viewed as a biological language, where gene expression profiles act as semantic tokens describing tissue molecular states [130], aligning histology images with transcriptomic signals, enabling cross-modal representation learning and downstream prediction [13, 16, 61, 74].

BLEEP [16] introduced the first multimodal framework. Its core idea is to maximize similarity between matched image-expression pairs while minimizing similarity between mismatched pairs. During inference, gene expression is predicted by retrieving the most similar known spots. Formally, for the $i$th spot consisting of an image $I_{i}$ and its corresponding gene expression vector $x_{i}$, the embeddings are obtained through an image encoder $f(\cdot )$ and a gene expression encoder $g(\cdot )$: 


(2) \begin{align*}& z_{i}^{\mathrm{img}} = f(I_{i}), \quad z_{i}^{\mathrm{gene}} = g(x_{i}),\end{align*}

The contrastive learning objective is based on a symmetric InfoNCE loss, which jointly optimizes image-to-gene and gene-to-image alignment: 


(3) \begin{align*} \mathcal{L}_{\mathrm{InfoNCE}} = \ & - \frac{1}{N} \sum_{i=1}^{N} \left[ \log \frac{\exp(\mathrm{sim}(z_{i}^{\mathrm{img}}, z_{i}^{\mathrm{gene}})/\tau)} {\sum_{j=1}^{N} \exp(\mathrm{sim}(z_{i}^{\mathrm{img}}, z_{j}^{\mathrm{gene}})/\tau)} \right.\nonumber \\ & \left.+ \log \frac{\exp(\mathrm{sim}(z_{i}^{\mathrm{gene}}, z_{i}^{\mathrm{img}})/\tau)} {\sum_{j=1}^{N} \exp(\mathrm{sim}(z_{i}^{\mathrm{gene}}, z_{j}^{\mathrm{img}})/\tau)} \right]\end{align*}

where $\mathrm{sim}(\cdot , \cdot )$ denotes cosine similarity and $\tau $ is a temperature parameter.

During inference, BLEEP predicts gene expression by comparing the embedding of a query image with all gene expression embeddings in a reference database. Given a query image $I_{q}$ with embedding $z_{q}^{\mathrm{img}}$, the similarity scores with all database embeddings $\{z_{j}^{\mathrm{gene}}\}_{j=1}^{M}$ are computed as: 


(4) \begin{align*}& s_{j} = \mathrm{sim}(z_{q}^{\mathrm{img}}, z_{j}^{\mathrm{gene}}).\end{align*}

The top-$K$ most relevant gene expression vectors $\{x_{j_{k}}\}_{k=1}^{K}$ are then retrieved, and the final prediction is obtained by a weighted aggregation: 


(5) \begin{align*}& \hat{y}_{q} = \sum_{k=1}^{K} w_{k} \, x_{j_{k}}, \quad w_{k} = \frac{\exp(s_{j_{k}}/\tau)}{\sum_{l=1}^{K} \exp(s_{j_{l}}/\tau)}.\end{align*}

Building on this framework, mclSTExp [61] extends BLEEP by incorporating spatial coordinates for joint spatial modeling. Meanwhile, as ST data scale grows, cross-modal alignment has expanded beyond image-gene expression to richer modalities such as image-ST gene sentence. These extensions improve semantic representation and create new opportunities for vision-omics FM [13].

#### Generation-based paradigm

The generation-based paradigm attempts to directly model the joint distribution between histology images and gene expression [46, 68, 71, 73]. In recent years, the emergence of diffusion models [131] has provided new opportunities for this task. STEM [68] first incorporates histology images as conditional variables, using DiT [132] as the backbone to reconstruct gene expression matrices in a generative manner. By modeling the probabilistic generation process, STEM captures complex distributions and provides stable and diverse outputs. Formally, let a gene expression vector be $x_{0} \in \mathbb{R}^{G}$, where $G$ denotes the number of genes. The forward diffusion process is defined as: 


(6) \begin{align*}& q(x_{t} \mid x_{0}) = \mathcal{N}\!\left(x_{t}; \sqrt{\alpha_{t}} \, x_{0}, (1-\alpha_{t}) I \right), \quad t=1,\dots,T\end{align*}

where $x_{t}$ denotes the noisy sample at step $t$, and $\{\alpha _{t}\}_{t=1}^{T}$ is the noise schedule.

In the reverse generation process, the model learns the conditional distribution: 


(7) \begin{align*}& \begin{aligned} p_\theta(x_{t-1} \mid x_{t}, I) &= \mathcal{N}\!\Bigl(x_{t-1}; \mu_\theta(x_{t}, t, h(I)), \\ & \Sigma_\theta(x_{t}, t, h(I)) \Bigr) \end{aligned}\end{align*}

where $I$ denotes the histology image and $h(I)$ is the conditional embedding extracted by an image encoder (e.g. a DiT backbone). Training is typically performed by minimizing the noise prediction objective, equivalent to a variational lower bound: 


(8) \begin{align*}& \mathcal{L}_{\mathrm{diff}} = \mathbb{E}_{x_{0}, \epsilon, t} \Big[ \| \epsilon - \epsilon_\theta(x_{t}, t, h(I)) \|^{2} \Big]\end{align*}

where $\epsilon \sim \mathcal{N}(0, I)$ denotes Gaussian noise, and $\epsilon _\theta $ is the noise predicted by the model. During inference, given a histology image $I$ as a condition, the diffusion model starts from Gaussian noise $x_{T} \sim \mathcal{N}(0, I)$ and progressively applies the conditional denoising process to generate the final gene expression prediction $\hat{x}_{0}$.

More recently, STFlow [71] introduces Flow Matching [133], a novel generative modeling framework that directly learns a deterministic, time-dependent vector field $v_\theta (x,t)$. STFlow uses histology images as conditional inputs, where the conditional embedding $h(I)$ guides the deterministic transport from a Gaussian prior to the gene expression distribution: 


(9) \begin{align*}& \frac{dx}{dt} = v_\theta(x, t, h(I)).\end{align*}

Thus, flow Matching reduces the number of sampling steps and improves both the stability of the generated results.

## Results

Differences in data preprocessing, gene selection, and training and validation schemes lead to diverse experimental settings. In this section, we review existing models from these three aspects and summarize commonly used evaluation metrics. Table 5 presents the implementation strategies of existing methods and their performance on specific datasets.

**Table 5:** Comparison of vision-to-ST models across datasets, preprocessing pipelines, validation strategies, and performance, with all metrics taken directly from the original papers.

**Model**	**Dataset**	**Preprocessing**	**Validation strategy**	**Cross-Val**	**Gene selection**	**MSE**	**PCC**
TRIPLEX [50]	HER2 [93]	Library Log-norm	Slide-level	$\checkmark $	HEG 250	0.228	0.497
His2ST [18]	HER2 [93]	Library Log-norm	Leave One Section Out	$\checkmark $	HVG 785	–	0.155
THItoGene [17]	HER2 [93]	Library Log-norm	Leave One Section Out	$\checkmark $	HVG 785	–	0.187
mclSTExp [61]	HER2 [93]	Library Log-norm	Leave One Section Out	$\checkmark $	HEG 50	0.589	0.388
ASIGN [14]	HER2 [93]	Library Log-norm	Sample-level	$\checkmark $	HEG 250	0.363	0.693
M2ORT [47]	HER2 [93]	Library Log-norm	Slide-level		HVG 250	0.029	0.443
Stem [68]	HER2 [93]	Log	Leave One Section Out	–	HVG 300	1.074	0.598
ST-Net [8]	Breast-ST [8]	Library Log-norm	Leave One Section Out	$\checkmark $	HEG 5	–	0.482
TRIPLEX [50]	Breast-ST [8]	Library Log-norm	Slide-level	$\checkmark $	HEG 250	0.202	0.352
EGN [15]	Breast-ST [8]	Log-Norm(min-max)	–	–	HEG 250	0.041	0.202
EGGN [43]	Breast-ST [8]	Log-Norm(min-max)	–	–	HEG 250	0.039	0.292
ASIGN [14]	Breast-ST [8]	Library Log-norm	Sample-level	$\checkmark $	HEG 250	0.332	0.739
M2ORT [47]	Breast-ST [8]	Library Log-norm	Slide-level	–	HVG 250	0.031	0.485
mclSTExp [61]	cSCC [92]	Library Log-norm	Leave One Section Out	$\checkmark $	HEG 50	0.233	0.361
TRIPLEX [50]	cSCC [92]	Library Log-norm	Slide-level	$\checkmark $	HEG 250	0.268	0.490
M2ORT [47]	cSCC [92]	Library Log-norm	Slide-level	–	HVG 250	0.033	0.509
BLEEP [16]	Human-Liver [101]	Log	Leave One Section Out	$\checkmark $	HVG 50	–	0.173
Stem [68]	Human-Kidney [94]	Log	Leave One Section Out		HVG 300	1.351	0.425

### Implementation for generative spatial omics

#### Gene selection

Owing to the sparsity of ST expression data, early studies [8, 14, 15] often selected highly expressed genes (HEGs) as prediction targets [8]. However, this strategy may overlook important spatial information. To better capture the link between tissue structure and molecular states, many later methods [16, 68] instead use highly variable genes (HVGs), improving sensitivity to spatial heterogeneity.

Gene selection scale also varies substantially across methods. Some studies [16, 61] focus on a small set of key genes (e.g. 50 targets) for specific pathways or downstream tasks, while others [18, 128] extend prediction to hundreds of genes (up to 785) to cover the transcriptomic space more broadly, reflecting different trade-offs between biological interpretability and computational complexity.

#### Preprocessing

Existing gene expression preprocessing methods can generally be grouped into two categories. The first directly applies logarithmic transformation to raw counts to reduce the long-tail effect in ST data [16, 68], thereby smoothing differences between low- and high-expression genes. The second first normalizes raw counts and then applies log transformation [8, 14, 43, 47, 50].

In practice, the second strategy has gradually become the mainstream choice, ensuring comparability of expression values across different spots, compresses the dynamic range of the distribution. By combining library size normalization with log transformation, it not only reduces the influence of extreme values but also alleviates biases caused by differences in sequencing depth. Specifically, let $x_{i,j}$ denotes the raw expression of gene $j$ in spot $i$. The Library size normalization is performed as: 


(10) \begin{align*}& x^{\mathrm{norm}}_{i,j} = \frac{x_{i,j}}{\sum_{k} x_{i,k}} \times s,\end{align*}

where $\sum _{k} x_{i,k}$ represents the total expression (library size) of spot $i$, and $s$ is a scaling factor (commonly $10^{4}$ or $10^{6}$). Subsequently, a log transformation is applied: 


(11) \begin{align*}& x^{\mathrm{log-norm}}_{i,j} = \log \bigl(1 + x^{\mathrm{norm}}_{i,j}\bigr).\end{align*}

In addition to conventional numerical preprocessing, some methods [21, 109] further discretize the raw counts into bins to mitigate differences in gene expression across cohorts. This binning strategy reduces data sparsity and noise, while also providing a standardized input representation for training large-scale FM.

#### Validation strategy

Different methods use substantially different ST data split strategies. Early studies [8, 50] commonly adopted slide-level cross-validation, treating different sections from the same sample as independent training and validation units. Another widely used strategy is Leave-One-Section-Out [16, 47, 128], where one section is reserved for validation and the others are used for training. However, because ST data often come from adjacent layers or regions of the same sample, these sections usually share similar tissue morphology and molecular patterns, which can inflate reported performance.

To address this issue, recent studies have introduced stricter splitting strategies. Some [14] use sample-level splits, ensuring that all sections from the same sample belong entirely to either training or validation. Others [50] employ external validation on independent datasets to provide a more objective assessment of generalization. Recent work [134] has also begun to systematically benchmark existing methods under more unified and fair settings.

### Evaluation metrics

We summarized current popular evaluation metrics in Table 6.

**Table 6:** Common evaluation metrics used in vision-to-ST tasks, grouped by category together with their corresponding formulas.

**Metric**	**Category**	**Formula**
MSE	Error	$ \tfrac{1}{n} \sum _{i=1}^{n} (y_{i} - \hat{y}_{i})^{2} $
MAE	Error	$ \tfrac{1}{n} \sum _{i=1}^{n} \lvert y_{i} - \hat{y}_{i} \rvert $
PCC	Correlation	$ \tfrac{\sum _{i=1}^{n} (y_{i} - \bar{y})(\hat{y}_{i} - \bar{\hat{y}})}{\sqrt{\sum _{i=1}^{n} (y_{i} - \bar{y})^{2}}\,\sqrt{\sum _{i=1}^{n} (\hat{y}_{i} - \bar{\hat{y}})^{2}}} $
SSIM	Correlation	$ \tfrac{(2\mu _{x} \mu _{y} + c_{1})(2\sigma _{xy} + c_{2})}{(\mu _{x}^{2} + \mu _{y}^{2} + c_{1})(\sigma _{x}^{2} + \sigma _{y}^{2} + c_{2})} $
RVD	Variation	$ \tfrac{1}{C} \sum _{j=1}^{C} \tfrac{(\sigma _{\mathrm{pred}}^{2,j} - \sigma _{\mathrm{gt}}^{2,j})^{2}}{(\sigma _{\mathrm{gt}}^{2,j})^{2}} $
ARI	Clustering	$ \mathrm{ARI} = \frac{\mathrm{Index} - \mathrm{Expected}}{\mathrm{Max} - \mathrm{Expected}} $
NMI	Clustering	$ \tfrac{2 \cdot I(U;V)}{H(U) + H(V)} $

$y_{i},\hat{y}_{i}$ denote ground-truth and prediction; $\bar{y},\bar{\hat{y}}$ are their means.$\mu _{x},\mu _{y},\sigma _{x}^{2},\sigma _{y}^{2},\sigma _{xy}$ are local statistics; $c_{1},c_{2}$ are constants.$\sigma _{\mathrm{pred}}^{2,j},\sigma _{\mathrm{gt}}^{2,j}$ are variances of gene $j$; $C$ is the number of genes.$n_{ij}$ is the contingency table entry, with row sums $n_{i\cdot }$, column sums $n_{\cdot j}$, and $n$ the total number of samples. ARI can equivalently be computed as: $\mathrm{ARI} = \tfrac{\sum _{ij}{{n_{ij}}\choose{2}} - \big [\sum _{i} {{n_{i\cdot }}\choose{2}}\sum _{j} {{n_{\cdot j}}\choose{2}}\big ]/{{n}\choose{2}}}{\tfrac{1}{2}\big [\sum _{i} {{n_{i\cdot }}\choose{2}} + \sum _{j} {{n_{\cdot j}}\choose{2}}\big ] - \big [\sum _{i} {{n_{i\cdot }}\choose{2}}\sum _{j} {{n_{\cdot j}}\choose{2}}\big ]/{{n}\choose{2}}}$.$I(U;V)$ is mutual information, $H(\cdot )$ entropy.

Mean squared error (MSE) and mean absolute error (MAE) are the most widely used metrics in ST prediction [8, 14, 50], mainly for regression tasks that predict gene expression levels. MSE is more sensitive to large errors, whereas MAE is more robust to outliers. The Pearson Correlation Coefficient (PCC) measures the linear correlation between predicted and ground-truth expression profiles and is commonly used to assess whether models capture spatial expression trends [8, 48, 128]. However, PCC can be unstable in high-resolution sparse data. In contrast, the Structural Similarity Index (SSIM), originally developed for image quality assessment, evaluates whether predicted spatial maps preserve structural patterns consistent with morphology [62, 73, 135].

Relative variation distance (RVD) measures the difference between predicted and ground-truth variance structures at the gene level [68]. It complements PCC by assessing whether a model preserves biological heterogeneity rather than producing near-mean predictions [16, 68]. For clustering tasks such as spatial domain identification, adjusted rand index (ARI) and normalized mutual information (NMI) are also commonly used [12, 39, 59]. ARI evaluates the consistency between predicted and true cluster assignments, while NMI measures the shared information between the two partitions, together reflecting the ability to capture cellular heterogeneity.

### Visual interpretability for vision-omics models

Most existing studies [8, 14, 136] focus on predictive performance, typically measured by PCC and MSE, while giving limited attention to whether predictions are grounded in meaningful morphological regions. However, morphological plausibility is important for transparency in ST analysis. Although methods such as HisToGene [40] and conST [41] provide preliminary visualizations, these remain auxiliary rather than systematic explainable AI analyses. In this section, we review current visual interpretability strategies for vision-omics models and discuss the limitations of existing XAI approaches in ST analysis.

#### Attention map in transformer-based methods

For Transformer-based models [137], attention maps are a widely used interpretability tool. HisToGene [40] shows a shift from local cellular morphology in shallow layers to broader tissue context in deeper layers. Later studies extend this idea: SPiRiT [138] links high-attention regions to marker genes, while PathCLAST [139] aggregates attention over a gene–pathway graph to estimate contributions. However, attention varies more across layers than across genes [140], suggesting that highlighted regions may reflect hierarchical tissue context rather than gene-specific cues. Therefore, attention maps are informative but do not by themselves ensure gene-level semantic interpretability.

#### Gradient-based attribution in CNN and hybrid models

For CNN-based or hybrid models, gradient-based methods such as Grad-CAM [141] and Integrated Gradients [142] provide complementary visual explanations. A recent Grad-CAM study [143] found that activation maps were nearly identical across different predicted genes, including CD4 and PDGFRA, despite their distinct spatial patterns, suggesting reliance on generic tissue-region importance rather than gene-specific morphology–molecule relationships. Earlier work such as HE2RNA [144] addressed this issue by generating gene-specific tile-level heatmaps and validating them with IHC staining, achieving a Pearson correlation of 0.51 for CD3D/E/G. Together, these studies show that gradient-based XAI can reveal not only where models attend, but also whether they truly capture biologically meaningful regions.

#### Post-hoc XAI

Beyond model-specific interpretability methods, *post-hoc* XAI approaches such as LIME [145] and SHAP [146] provide a unified explanation framework for both CNN- and Transformer-based systems by perturbing inputs and measuring prediction changes. STimage [147] combines Cellpose-based segmentation with LIME to quantify nuclear-level contributions to gene prediction. SpaPheno [148] computes SHAP values at the spot level to localize tissue regions and cell types driving clinical phenotype prediction. HistoTME [149] further integrates attention heatmaps and SHAP summary plots to identify tumor microenvironment features predictive of immunotherapy response. Overall, *post-hoc* methods offer model-agnostic flexibility, but require substantially higher computational cost, especially for high-resolution images and large gene panels.

## Discussion

### Emerging methodological directions

Beyond mainstream supervised and multimodal representation learning, ST is expanding toward broader computational paradigms, as summarized in Table 7. Three directions are particularly notable, including RL for experimental design, agentic AI for workflow automation, and FM for cross-organ generalization, reflecting a broader methodological shift to considering what to measure, how to analyze, and what to validate next.

**Table 7:** Landscape of emerging methodological directions in ST (2024–2026), with checkmarks indicating the primary paradigm of each model, including FM, RL, and agent-based methods.

**Model**	**Year**	**FM**	**RL**	**Agent**
QuST-LLM [118]	2024-June			$\checkmark $
SOFisher [116]	2024-July		$\checkmark $	
scGPT-spatial [109]	2025-February	$\checkmark $		
STAgent [120]	2025-April			$\checkmark $
OmiCLIP [13]	2025-May	$\checkmark $		
HEIST [110]	2025-June	$\checkmark $		
SToFM [111]	2025-July	$\checkmark $		
spaLLM [150]	2025-July			$\checkmark $
SpaFoundation [112]	2025-August	$\checkmark $		
FmH2ST [113]	2025-September	$\checkmark $		
STPath [84]	2025-November	$\checkmark $		
S2-omics [117]	2025-November		$\checkmark $	
SCR$^{2}$-ST [87]	2025-December		$\checkmark $	
spEMO [114]	2026-February	$\checkmark $		
ChatSpatial [121]	2026-February			$\checkmark $
EnsAgent [122]	2026-March			$\checkmark $

#### Reinforcement learning for data acquisition

Classical RL is still relatively uncommon in ST, mainly because it requires well-defined states, actions, and rewards, which are difficult to construct for high-dimensional gene expression data with sparse supervision and delayed feedback. SOFisher [116] formulates field-of-view selection as a sequential decision-making problem, using an RL agent to identify imaging regions that maximize biological information while reducing sequencing cost. SCR$^{2}$-ST [87] applies RL-guided active sampling to prioritize informative spatial locations under limited sequencing budgets. S2-omics [117] further extends this idea to multimodal spatial omics, using H&E-guided region-of-interest selection to determine where high-resolution profiling should be performed. Overall, RL provides a principled way to prioritize tissue regions, spatial locations, and modalities for measurement. This role is complementary to supervised prediction and may become crucial as higher-resolution spatial platforms continue to raise experimental costs.

#### Agentic artificial intelligence for automated spatial analysis

Agentic AI, especially LLM-based tool-using systems, is advancing rapidly in ST. This is largely because ST analysis itself involves multi-step workflows. Such workflows are naturally suited to agent-based orchestration, where a language model can dynamically select and coordinate analysis tools according to the biological question. CellAgent [119] exemplifies this paradigm by enabling end-to-end single-cell and ST analysis through natural-language interaction, achieving expert-level performance on many standard tasks. STAgent [120] further tailors this idea to ST by integrating quality control, spatial domain identification, and cell–cell communication analysis into a unified interactive system. stMCP [123] takes a more modular approach by exposing ST analysis functions as callable tools for MCP-compatible LLM agents, facilitating integration with broader agent ecosystems. Together, these studies suggest that agentic systems in spatial omics are developing less through RL-style policy optimization and more through LLM-driven workflow automation, tool coordination, and biological reasoning.

#### Foundation models for spatial transcriptomics

Foundation models are emerging as one of the most transformative directions in ST. Unlike task-specific supervised models trained on individual datasets, they aim to learn transferable representations from large-scale data, enabling zero-shot or few-shot generalization across organs, platforms, and experimental settings. Broadly, the current landscape includes cross-modal vision-omics FM, H&E-to-ST prediction FM, and ST-native FM.

The first group aligns images with transcriptomic profiles through large-scale pretraining. OmiCLIP [13] first established this direction, enabling zero-shot tissue annotation and cross-modal retrieval, while SEAL [115] further pushes this paradigm toward clinical deployment by adapting pathology FM with ST supervision and requiring only H&E images at inference. The second group focuses on large-scale H&E-to-ST prediction. STPath [84] and SpaFoundation [112] shows that image-only pretraining can already support competitive zero-shot ST prediction. The third group consists of ST-native FM, which pretrain directly on spatial gene expression and cell graphs without relying on histology images. Representative examples such as SToFM [111], stFormer [59], and scGPT-spatial [109] learn multi-scale spatial representations from large transcriptomic corpora, improving downstream tasks such as clustering, interaction modeling, and missing gene imputation.

While substantial progress has been achieved in data resources and modeling paradigms, several key challenges remain. In this section, we discuss these pressing issues and further outline potential learning strategies and opportunities, providing a roadmap for the next phase of vision–omics integration.

### Multimodal data integration in vision-omics modeling

Multimodal data integration [13, 16, 61] has become a major direction in ST because no single modality can fully capture tissue biology. Histopathology images provide rich morphological information, whereas gene expression profiles reveal molecular states but lack direct morphological context. By integrating these complementary modalities, multimodal approaches establish a stronger link between tissue phenotype and molecular identity, enabling biological insights that neither modality can provide alone.

#### Functional benefits of multimodal fusion

Multimodal fusion mainly enhances biological interpretability and predictive robustness. By jointly modeling tissue morphology and molecular readouts, these frameworks capture cross-modal relationships that are difficult to recover from either modality alone. For example, BLEEP [16] aligns H&E images and gene expression in a shared embedding space and improves marker gene prediction over unimodal methods such as ST-Net [8], while OmiCLIP [13] extends this paradigm to 2.2 million paired samples across 32 organs for zero-shot tissue annotation. HisToGene [40] likewise shows the value of joint modeling for recovering tissue heterogeneity. Multimodal fusion also improves robustness, since one modality can compensate for the sparsity, noise, or limited resolution of the other. For instance, STAIG [19] and SpaICL [77] use image-derived features to guide spatial graph construction, reducing reliance on raw expression quality. This complementary effect is particularly valuable in clinical settings with variable staining quality, tissue preservation, and sequencing depth.

#### Structural barriers to reliable cross-modal alignment

The key limitation of current multimodal ST models is not only imperfect performance, but also that higher accuracy may still fail to indicate genuine gene-specific understanding. In practice, many models seem to identify globally informative tissue regions rather than learn distinct morphology-molecule correspondences for different genes [143]. This gap reflects several structural barriers to reliable cross-modal alignment.

A major challenge is resolution mismatch. H&E images provide continuous visual information at sub-micron resolution, whereas most ST platforms measure gene expression at the spot level ($\sim $55 $\mu $m), with each spot mixing signals from multiple cell types. This discrepancy sets an inherent limit on alignment precision. GHIST [79] begins to address this issue with subcellular-resolution ST supervision, and FineST [151] further pushes alignment to the nuclei level through segmentation and contrastive learning. However, the impact of intra-spot cellular heterogeneity on cross-modal alignment remains insufficiently quantified.

Reference set bias is another barrier, especially in retrieval-based frameworks. Methods such as BLEEP [16] rely heavily on the composition of the reference library, so predictions can be biased toward overrepresented platforms, tissues, or disease types. Large-scale pretraining in OmiCLIP [13] partially alleviates this issue, but current public ST datasets remain strongly imbalanced in platform and disease distribution.

Computational complexity is also a major constraint. Joint training of pathology FM and transcriptomic encoders is often too expensive on standard hardware, forcing most methods to operate at spot-level resolution. As a result, the practical resolution of multimodal fusion is often limited more by computational budget than by biological need. Lightweight alternatives, such as graph-based methods [19, 77] or contrastive heads on frozen backbones [61], offer partial relief but usually with reduced expressive capacity.

### Data processing

A major challenge for vision-driven ST models lies at the data level. High-quality deep learning models require abundant and reliable ST data, yet such resources remain limited. In addition, substantial heterogeneity across sequencing platforms in capture efficiency and sequencing depth [98] leads to differences in data format and signal strength, complicating cross-platform comparison and model transfer [152]. Another difficulty is the imperfect spatial alignment between histopathology images and molecular signals. Misalignment can arise from section deformation or tissue cutting offsets [153, 154], while batch effects and differences in sample preparation can further obscure biological signals or introduce spurious correlations. In some cases, even conventional library-size normalization may impair spatial domain identification [155].

Addressing these issues requires progress in data integration and standardization. Larger and more balanced datasets [99, 100] are needed to reduce distributional bias, while unified preprocessing and analysis pipelines [156, 157], including cross-platform standardization and batch-effect correction, are essential for comparability across sources.

### Fair and reproducible pipeline

Existing studies still lack unified and fair training-validation protocols. First, data partitioning is inconsistent. Many works use leave-one-section-out [16, 128] or slide-level random splits [47, 50], which often place highly correlated samples from the same patient or tissue in both training and test sets, causing information leakage and inflated performance. In contrast, patient- or center-level splits better reflect clinically meaningful external generalization [14].

Second, most methods are evaluated on only a single dataset, with limited cross-dataset and cross-platform testing [50]. As a result, robustness to distribution shift, batch effects, and experimental variation remains unclear. In addition, differences in test-gene sets and preprocessing pipelines, including gene selection, normalization, and quality control, can affect reported metrics and distort fair comparison.

Recent efforts have begun to establish more equitable and reproducible evaluation frameworks [134]. Larger cross-cohort benchmarks [99, 100], together with standardized partition schemes, gene selection, and preprocessing, are needed to avoid performance artifacts caused by split and pipeline choices. At the same time, open-sourcing code and weights, fixing dependencies and random seeds, and providing one-click evaluation pipelines will improve reproducibility and transparency. In this way, vision-driven ST research can move beyond “works on this dataset” toward cross-dataset, cross-platform, and deployment-ready standards.

### Towards robust evaluation

Current evaluation protocols remain a major bottleneck in vision-based ST prediction. Most studies still rely mainly on numerical error metrics such as MSE and MAE, which primarily reflect curve-fitting ability but provide limited insight into whether a model captures biologically meaningful spatial patterns or gene-specific morphology–molecule associations. Emerging XAI evidence suggests that a model may achieve strong numerical performance while behaving largely as a generic tissue-region scorer rather than a true interpreter of molecularly relevant histomorphology. In addition, correlation-based metrics such as PCC also have important limitations, as they can become unstable in highly sparse data [73] or under mean-like prediction behavior [68], thereby misrepresenting actual model quality.

Future evaluation should move toward standardization. Beyond conventional regression metrics, benchmarks should incorporate spatial and biological criteria, such as marker–gene recovery, spatial pattern preservation, and region-level consistency, to better reflect biological interpretability and fidelity. More importantly, mechanism-oriented metrics are needed to assess whether models recover biologically meaningful structure, including pathway-level recovery and disease-relevant marker detectability. Such a framework would shift evaluation from numerical fit alone toward biological validity, enabling a more faithful assessment of model value for interpretation and clinical translation.

### Applications and potential learning strategies

Existing vision-driven ST studies most still focus on foundational tasks and with limited progress toward clinical translation. Bridging basic research and clinical practice, therefore, remains a key challenge [158, 159]. Moreover, the lack of a unified framework for multimodal integration hinders building more comprehensive tissue representations [13]. With the emergence of large-scale standardized resources such as HEST-1k [99] and STImage-1K4M [100], together with advances in RL, and agentic AI, new opportunities are emerging. In this section, we discuss three application directions and the learning strategies that may support them.

#### Scaling generalization and data re-usability

A practical direction is to improve model applicability across institutions, platforms, and cohorts. Early methods usually relied on a single data source [8, 16], and their performance often dropped markedly when transferred to different centers or experimental settings [160]. Addressing this issue will likely require combining large-scale cross-organ and cross-platform pretraining [13, 84] with parameter-efficient adaptation strategies [115], where the former provides more diverse tissue representations and the latter enables rapid adaptation to new environments and technical platforms. Yet, cross-institutional analysis still involves labor-intensive steps such as preprocessing, quality control, and result integration.

LLM-based agentic systems [119, 120] offer a complementary solution by automating workflows through natural-language interaction, potentially reducing the burden of large-scale multi-center analysis. At the same time, the molecular inference capacity of H&E images continues to expand, from transcriptomic prediction to spatial protein biomarker inference [161, 162] and even population-scale virtual tumor microenvironment modeling [163, 164]. This suggests that large pathology slide archives can be further mined for rich multi-omics features without additional wet-lab assays, offering clear value for retrospective studies and resource-limited settings.

#### Spatial molecular modeling under data limitation and expertise constraints

Cross-institutional generalization addresses how existing data can be better reused, but in many clinical settings the more fundamental challenge is data scarcity. Rare diseases and small clinical subpopulations remain difficult to study because task-specific, well-annotated training data are limited. Emerging strategies are beginning to reduce this dependence. Few-shot transfer learning allows FM to adapt from limited annotations [165], parameter-efficient fine-tuning achieves competitive performance by updating only a small fraction of parameters [115, 166], and zero-shot cross-modal generalization extends transfer to molecular modalities not explicitly seen during training [110]. At the same time, agentic systems may lower not the data barrier but the expertise barrier. Through natural-language interaction, they can help researchers without extensive computational backgrounds perform end-to-end workflows, from data preprocessing to biological interpretation [119, 123]. Together, these advances may make biological questions once limited by cohort size or technical barriers increasingly accessible to systematic study.

#### Intelligent and adaptive spatial omics data acquisition

The previous discussion focused on how to better use existing data, but a more fundamental question is whether data acquisition itself can be made more efficient. Conventional spatial omics experiments usually follow a broad profiling paradigm, in which tissues are comprehensively measured first and regions or genes are selected only afterward for deeper analysis. This workflow is resource-intensive and will become even more costly as higher-resolution platforms continue to increase the expense of spatial omics experiments.

The convergence of large-scale pretraining, RL, and agentic AI is beginning to support an alternative paradigm. Screening before measurement and targeting before profiling. Foundation models enable prescreening of tissue sections before spatial molecular assays to identify high-value regions and candidate biomarkers [13, 112]. Reinforcement learning provides a principled way to decide where and what to measure under limited budgets [87, 116, 117], while agentic systems may connect prescreening, decision-making, and interpretation into an automated workflow [120, 123]. In this way, spatial omics are moving from exhaustive measurement followed by *post hoc* selection to targeted judgment.

### Limitations

This review focuses on vision-driven models for ST published between 2020 and 2025. Although we aimed to cover the most representative advances, our selection is inevitably limited by publication timing and the availability of public evaluations, so some very recent or insufficiently benchmarked studies may not be included. In addition, both computer vision and ST are evolving rapidly, with new methods and paradigms continually reshaping the field. Therefore, this article should be regarded as a time-bounded overview rather than a definitive account, and readers are encouraged to follow subsequent studies, datasets, and emerging evidence to complement and update its coverage.

## Conclusion

In this survey, we systematically review representative advances in vision-driven models for ST analysis from multiple perspectives, including model architectures, learning paradigms, downstream tasks, and data resources. Vision-driven ST has moved beyond early regression-based methods toward multimodal and generative frameworks, with applications expanding from gene expression prediction and clustering to more complex tasks such as 3D reconstruction. This trend reflects both methodological diversification and growing practical potential. At the same time, the field still faces major challenges, including the scarcity and heterogeneity of high-quality datasets, the lack of standardized and fair evaluation protocols, and limited cross-platform generalization. These issues indicate that vision-driven ST remains in a stage of rapid development. Looking ahead, further progress will require more efficient model architectures, deeper multimodal integration, and larger high-quality benchmark datasets. Overall, the evolution of vision-driven ST represents an important frontier at the intersection of bioinformatics and medical imaging, offering new opportunities to understand tissue organization and disease mechanisms while bridging basic research and clinical application.

**Key Points**

- This review provides the first comprehensive and systematic overview of existing vision-driven spatial transcriptomics (ST) models, covering methodological foundations, core architectures, and downstream applications in a structured and hierarchical manner.
- Key focus is placed on the taxonomy and comparative analysis of current models across different training paradigms and real-world application scenarios, emphasizing their architectural designs, training strategies, data requirements, and task suitability.
- Given the growing complexity and diversity of vision-driven ST frameworks, we establish clear connections among research directions and delineate how model evolution reflects shifts in computational and biological perspectives.
- We further discuss open challenges and future research directions in vision-driven spatial transcriptomics, highlighting key bottlenecks, emerging trends, and potential avenues toward interpretable, scalable, and multimodal integration frameworks.

## Data Availability

The datasets referenced in this review can be accessed through the original publications cited in the manuscript. The curated collection of vision-driven spatial transcriptomics papers is available at https://github.com/hrlblab/computer_vision_spatial_omics.

## References

[1] Asp M, Bergenstråhle J, Lundeberg J. Spatially resolved transcriptomes—next generation tools for tissue exploration. *BioEssays* 2020;42:e1900221. 10.1002/bies.201900221 32363691

[2] Burgess DJ . Spatial transcriptomics coming of age. *Nat Rev Genet* 2019;20:317–7. 10.1038/s41576-019-0129-z 30980030

[3] Asp M, Giacomello S, Larsson L et al. A spatiotemporal organ-wide gene expression and cell atlas of the developing human heart. *Cell* 2019;179:1647–1660.e19. 10.1016/j.cell.2019.11.025 31835037

[4] Maniatis S, Äijö T, Vickovic S et al. Spatiotemporal dynamics of molecular pathology in amyotrophic lateral sclerosis. *Science* 2019;364:89–93. 10.1126/science.aav9776 30948552

[5] Cai J, Cheng H, Shushan W et al. West is an ensemble method for spatial transcriptomics analysis. *Cell Rep Methods* 2024;4:100886. 10.1016/j.crmeth.2024.100886 39515332 PMC11705770

[6] Pratama R, Jason Hilton J, Cherry M et al. Gene spatial integration: enhancing spatial transcriptomics analysis via deep learning and batch effect mitigation. *Bioinformatics* 2025;41:btaf350. 40511994 10.1093/bioinformatics/btaf350 PMC12208067

[7] Schott M, León-Periñán D, Splendiani E et al. Open-ST: high-resolution spatial transcriptomics in 3D. *Cell* 2024;187:3953–3972.e26. 10.1016/j.cell.2024.05.055 38917789

[8] He B, Bergenstråhle L, Stenbeck L et al. Integrating spatial gene expression and breast tumour morphology via deep learning. *Nat Biomed Eng* 2020;4:827–34. 10.1038/s41551-020-0578-x 32572199

[9] Ash JT, Darnell G, Munro D et al. Joint analysis of expression levels and histological images identifies genes associated with tissue morphology. Nat Commun 2021;12:1609. 10.1038/s41467-021-21727-x PMC7952575 33707455

[10] He K, Zhang X, Ren S et al. Deep residual learning for image recognition. In: Proceedings of the IEEE conference on computer vision and pattern recognition (CVPR). Las Vegas, NV, USA: IEEE; 2016, 770–8.

[11] Dosovitskiy A, Beyer L, Kolesnikov A et al. An image is worth 16x16 words: transformers for image recognition at scale. *arXiv preprint arXiv:2010.11929*, 2020.

[12] Pham D, Tan X, Xu J et al. stLearn: integrating spatial location, tissue morphology and gene expression to find cell types, cell-cell interactions and spatial trajectories within undissociated tissues. *biorxiv* 2020;2020–05.

[13] Chen W, Pengzhi Zhang TN, Tran YX et al. A visual–omics foundation model to bridge histopathology with spatial transcriptomics. Nat Methods 2025;22:1568–82. 10.1038/s41592-025-02707-1 PMC12240810 40442373

[14] Zhu J, Deng R, Yao T et al. ASIGN: an anatomy-aware spatial imputation graphic network for 3D spatial transcriptomics. In: Proceedings of the Computer Vision and Pattern Recognition Conference (CVPR). Nashville, TN, USA: IEEE/CVF; 2025, 30829–38.

[15] Yang Y, Hossain MZ, Stone EA et al. Exemplar guided deep neural network for spatial transcriptomics analysis of gene expression prediction. In: Proceedings of the IEEE/CVF Winter Conference on Applications of Computer Vision, (WACV). Waikoloa, HI, USA: IEEE/CVF; 2023, 5039–48.

[16] Xie R, Pang K, Chung S et al. Spatially resolved gene expression prediction from histology images via bi-modal contrastive learning. Adv Neural Inf Process Syst 2023;36:70626–37.

[17] Jia Y, Liu J, Chen L et al. THItoGene: a deep learning method for predicting spatial transcriptomics from histological images. *Brief Bioinform* 2024;25:bbad464. 10.1093/bib/bbad464 PMC10749789 38145948

[18] Zeng Y, Wei Z, Weijiang Y et al. Spatial transcriptomics prediction from histology jointly through transformer and graph neural networks. Brief Bioinform 2022;23:bbac297. 10.1093/bib/bbac297 35849101

[19] Yang Y, Cui Y, Zeng X et al. STAIG: spatial transcriptomics analysis via image-aided graph contrastive learning for domain exploration and alignment-free integration. *Nat Commun* 2025;16:1067. 10.1038/s41467-025-56276-0 39870633 PMC11772580

[20] Lais P, Mishra S, Xiong K et al. Image guided construction of a common coordinate framework for spatial transcriptome data. Sci Rep 2025;15:18074. 10.1038/s41598-025-01862-x PMC12103625 40413226

[21] Cui H, Wang C, Maan H et al. scGPT: toward building a foundation model for single-cell multi-omics using generative ai. *Nat Methods* 2024;21:1470–80. 10.1038/s41592-024-02201-0 38409223

[22] Lu MY, Chen B, Williamson DFK et al. A visual-language foundation model for computational pathology. *Nat Med* 2024;30:863–74. 10.1038/s41591-024-02856-4 38504017 PMC11384335

[23] Huang Z, Bianchi F, Yuksekgonul M et al. A visual–language foundation model for pathology image analysis using medical twitter. Nat Med 2023;29:2307–16. 10.1038/s41591-023-02504-3 37592105

[24] Marx V . Method of the year: Spatially resolved transcriptomics. *Nat Methods* 2021;18:9–14. 10.1038/s41592-020-01033-y 33408395

[25] Williams CG, Lee HJ, Asatsuma T et al. An introduction to spatial transcriptomics for biomedical research. Genome Med 2022;14:68. 10.1186/s13073-022-01075-1 PMC9238181 35761361

[26] Zhou R, Yang G, Zhang Y et al. Spatial transcriptomics in development and disease. *Mol Biomed* 2023;4:32. 10.1186/s43556-023-00144-0 37806992 PMC10560656

[27] Li X, Wang C-Y. From bulk, single-cell to spatial RNA sequencing. *Int J Oral Sci* 2021;13:36. 10.1038/s41368-021-00146-0 34782601 PMC8593179

[28] Rao A, Barkley D, França GS et al. Exploring tissue architecture using spatial transcriptomics. *Nature* 2021;596:211–20. 10.1038/s41586-021-03634-9 34381231 PMC8475179

[29] Moses L, Pachter L. Museum of spatial transcriptomics. *Nat Methods* 2022;19:534–46. 10.1038/s41592-022-01409-2 35273392

[30] Ståhl PL, Salmén F, Vickovic S et al. Visualization and analysis of gene expression in tissue sections by spatial transcriptomics. *Science* 2016;353:78–82. 10.1126/science.aaf2403 27365449

[31] Vickovic S, Eraslan G, Salmén F et al. High-definition spatial transcriptomics for in situ tissue profiling. *Nat Methods* 2019;16:987–90. 10.1038/s41592-019-0548-y 31501547 PMC6765407

[32] Xia C, Fan J, Emanuel G et al. Spatial transcriptome profiling by MERFISH reveals subcellular RNA compartmentalization and cell cycle-dependent gene expression. *Proc Natl Acad Sci USA* 2019;116:19490–9. 10.1073/pnas.1912459116 31501331 PMC6765259

[33] Eng C-HL, Lawson M, Zhu Q et al. Transcriptome-scale super-resolved imaging in tissues by RNA seqfish+. *Nature* 2019;568:235–9. 10.1038/s41586-019-1049-y 30911168 PMC6544023

[34] Qian X, Coleman K, Jiang S et al. Spatial transcriptomics reveals human cortical layer and area specification. Nature 2025;644:153–63. 10.1038/s41586-025-09010-1 PMC12328223 40369074

[35] Conacher B, Moore A, Liduo Yin Y et al. Spatial transcriptomics reveals regional and temporal dynamics of gene expression in the mouse brain across development and aging. *Biology* 2025;14:717. 10.3390/biology14060717 40563967 PMC12189371

[36] Jin Y, Zuo Y, Li G et al. Advances in spatial transcriptomics and its applications in cancer research. *Mol Cancer* 2024;23:129. 10.1186/s12943-024-02040-9 38902727 PMC11188176

[37] Shizhe Y, Wang H, Yang L et al. Spatial transcriptome profiling of normal human liver. *Sci Data* 2022;9:633. 10.1038/s41597-022-01676-w 36261431 PMC9581974

[38] Moncada R, Barkley D, Wagner F et al. Integrating microarray-based spatial transcriptomics and single-cell RNA-seq reveals tissue architecture in pancreatic ductal adenocarcinomas. *Nat Biotechnol* 2020;38:333–42. 10.1038/s41587-019-0392-8 31932730

[39] Jian H, Li X, Coleman K et al. SpaGCN: integrating gene expression, spatial location and histology to identify spatial domains and spatially variable genes by graph convolutional network. *Nat Methods* 2021;18:1342–51. 34711970 10.1038/s41592-021-01255-8

[40] Pang M, Kenong S, Li M. Leveraging information in spatial transcriptomics to predict super-resolution gene expression from histology images in tumors. *BioRxiv* 2021;2021–11.

[41] Zong Y, Tingyang Y, Wang X et al. conST: an interpretable multi-modal contrastive learning framework for spatial transcriptomics. *BioRxiv* 2022;2022–01.

[42] Monjo T, Koido M, Nagasawa S et al. Efficient prediction of a spatial transcriptomics profile better characterizes breast cancer tissue sections without costly experimentation. *Sci Rep* 2022;12:4133. 10.1038/s41598-022-07685-4 35260632 PMC8904587

[43] Yan Yang M, Hossain Z, Stone E et al. Spatial transcriptomics analysis of gene expression prediction using exemplar guided graph neural network. *Pattern Recogn* 2024;145:109966. 10.1016/j.patcog.2023.109966

[44] Rahaman MM, Millar EKA, Meijering E. Breast cancer histopathology image-based gene expression prediction using spatial transcriptomics data and deep learning. Sci Rep 2023;13:13604. 10.1038/s41598-023-40219-0 PMC10442349 37604916

[45] Mejia G, Cárdenas P, Ruiz D et al. SEPAL: Spatial gene expression prediction from local graphs. In: Proceedings of the IEEE/CVF International Conference on computer vision (ICCV). Paris, France: IEEE/CVF; 2023, 2294–303.

[46] Kumar S, Chatterjee S. HistoSPACE: histology-inspired spatial transcriptome prediction and characterization engine. *Methods* 2024;232:107–14. 10.1016/j.ymeth.2024.11.002 39521362

[47] Wang H, Du X, Liu J et al. M2ORT: many-to-one regression transformer for spatial transcriptomics prediction from histopathology images. *arXiv preprint arXiv:2401.10608*, 2024.

[48] Xiao X, Kong Y, Li R et al. Transformer with convolution and graph-node co-embedding: an accurate and interpretable vision backbone for predicting gene expressions from local histopathological image. *Med Image Anal* 2024;91:103040. 10.1016/j.media.2023.103040 38007979

[49] Zhao C, Zhongli X, Wang X et al. Innovative super-resolution in spatial transcriptomics: a transformer model exploiting histology images and spatial gene expression. *Brief Bioinform* 2024;25:bbae052. 38436557 10.1093/bib/bbae052 PMC10939304

[50] Chung Y, Ha JH, Im KC et al. Accurate spatial gene expression prediction by integrating multi-resolution features. In: Proceedings of the IEEE/CVF Conference on Computer Vision and Pattern Recognition (CVPR). Seattle, WA, USA: IEEE/CVF; 2024, 11591–600.

[51] Chen X, Lin J, Wang Y et al. HE2Gene: image-to-RNA translation via multi-task learning for spatial transcriptomics data. Bioinformatics 2024;40:btae343. 38837395 10.1093/bioinformatics/btae343 PMC11164830

[52] Zhong Y, Zhang J, Ren X. Spatial transcriptomics prediction from histology images at single-cell resolution using redehist. *bioRxiv* 2024;2024–06.

[53] Chi C, Shi H, Zhu Q, Zhang D et al. Spatially resolved gene expression prediction from histology via multi-view graph contrastive learning with hsic-bottleneck regularization. *arXiv preprint arXiv:2406.12229*, 2024.AU: Please provide the volume, page range, journal title for reference 53 directly in the reference.

[54] Jiang X, Wang S, Guo L et al. iIMPACT: integrating image and molecular profiles for spatial transcriptomics analysis. *Genome Biol* 2024;25:147. 10.1186/s13059-024-03289-5 38844966 PMC11514947

[55] Shi Z, Xue S, Zhu F et al. High-resolution spatial transcriptomics from histology images using histosge. In: *2024 IEEE International Conference on Bioinformatics and Biomedicine (BIBM)*. Lisbon, Portugal: IEEE; 2024, 2402–7.

[56] Zhang L, Liang S, Wan L. A multi-view graph contrastive learning framework for deciphering spatially resolved transcriptomics data. Brief Bioinform 2024;25:bbae255. 10.1093/bib/bbae255 PMC11129769 38801701

[57] Shi Z, Zhu F, Wang C et al. Spatial gene expression prediction from histology images with STco. In: International Symposium on Bioinformatics Research and Applications, vol 14955. Singapore: Springer; 2024, 89–100. 10.1007/978-981-97-5128-0_8

[58] Liu A, Zhao Y, Shen H et al. ResSAT: enhancing spatial transcriptomics prediction from H&E-stained histology images with interactive spot transformer. *Res Sq* 2024;rs–3. 10.1186/s13059-026-04168-x PMC13576212 42363230

[59] Cao S, Yang K, Cheng J et al. stFormer: a foundation model for spatial transcriptomics. *bioRxiv* 2024;2024–09.

[60] Li K, Li J, Tao Y et al. stDiff: a diffusion model for imputing spatial transcriptomics through single-cell transcriptomics. Brief Bioinform 2024;25:bbae171. 10.1093/bib/bbae171 PMC11021815 38628114

[61] Min W, Shi Z, Zhang J et al. Multimodal contrastive learning for spatial gene expression prediction using histology images. Brief Bioinform 2024;25:bbae551. 10.1093/bib/bbae551 PMC11952928 39471412

[62] Li B, Zhang Y, Wang Q et al. Gene expression prediction from histology images via hypergraph neural networks. Brief Bioinform 2024;25:bbae500. 10.1093/bib/bbae500 PMC11472757 39401144

[63] Pizurica M, Zheng Y, Carrillo-Perez F et al. Digital profiling of gene expression from histology images with linearized attention. *Nat Commun* 2024;15:9886. 10.1038/s41467-024-54182-5 39543087 PMC11564640

[64] Lin Y, Liang Y, Wang D et al. A contrastive learning approach to integrate spatial transcriptomics and histological images. *Comput Struct Biotechnol J* 2024;23:1786–95. 10.1016/j.csbj.2024.04.039 38707535 PMC11068546

[65] Niu J, Zhu F, Taosheng X et al. Deep clustering representation of spatially resolved transcriptomics data using multi-view variational graph auto-encoders with consensus clustering. *Comput Struct Biotechnol J* 2024;23:4369–83. 10.1016/j.csbj.2024.11.041 39717398 PMC11664090

[66] Lee Y, Liu X, Hao M et al. PathOmCLIP: connecting tumor histology with spatial gene expression via locally enhanced contrastive learning of pathology and single-cell foundation model. *bioRxiv* 2024;2024–12.

[67] Ge Y, Leng J, Tang Z et al. Deep learning-enabled integration of histology and transcriptomics for tissue spatial profile analysis. *Research* 2025;8:0568. 10.34133/research.0568 39830364 PMC11739434

[68] Zhu S, Zhu Y, Tao M et al. Diffusion generative modeling for spatially resolved gene expression inference from histology images. *arXiv preprint arXiv:2501.15598*, 2025.

[69] Zhang R, Yang Y, Pan L. Spatial transcriptomics analysis of spatially dense gene expression prediction. *arXiv preprint arXiv:2503.01347*, 2025.

[70] Zhu J, Deng R, Yao T et al. Magnet: multi-level attention graph network for predicting high-resolution spatial transcriptomics. *arXiv preprint arXiv:2502.21011*, 2025.

[71] Huang T, Liu T, Babadi M et al. Scalable generation of spatial transcriptomics from histology images via whole-slide flow matching. *arXiv preprint arXiv:2506.05361*, 2025. 10.1038/s41746-025-02020-3 PMC12618518 41238784

[72] Zhang T, Zhang H, Zhao Z et al. spaMGCN: a graph convolutional network with autoencoder for spatial domain identification using multi-scale adaptation. *Genome Biol* 2025;26:159. 10.1186/s13059-025-03637-z 40495225 PMC12150536

[73] Zhu J, Deng R, Guo J et al. Img2ST-Net: efficient high-resolution spatial omics prediction from whole slide histology images via fully convolutional image-to-image learning. *arXiv preprint arXiv:2508.14393*, 2025.

[74] Yan Q, Li X, Cui J et al. Spatial histology and gene-expression representation and generative learning via online self-distillation contrastive learning. Brief Bioinform 2025;26:bbaf317. 10.1093/bib/bbaf317 PMC12229093 40618351

[75] Yulong W, Xie J, Nie J et al. DANet: spatial gene expression prediction from H&E histology images through dynamic alignment. *Brief Bioinform* 2025;26:bbaf422. 40833275 10.1093/bib/bbaf422 PMC12365980

[76] Liu X, Jiang Z, Zhu P et al. HaDM-ST: histology-assisted differential modeling for spatial transcriptomics generation. In: International Workshop on Computational Mathematics Modeling in Cancer Analysis. Cham: Springer Nature Switzerland; 2025, 129–38. 10.1007/978-3-032-06624-4_14

[77] Zhao J, Min W. SpaICL: Image-guided curriculum strategy-based graph contrastive learning for spatial transcriptomics clustering. *Brief Bioinform* 2025;26:bbaf433. 40838787 10.1093/bib/bbaf433 PMC12368861

[78] Wang Y, Yang H, Deng R et al. StImage: a versatile framework for optimizing spatial transcriptomic analysis through customizable deep histology and location informed integration. *Brief Bioinform* 2025;26:bbaf429. 40905789 10.1093/bib/bbaf429 PMC12409783

[79] Xiaohang F, Cao Y, Bian B et al. Spatial gene expression at single-cell resolution from histology using deep learning with GHIST. Nat Methods 2025;22:1900–10. 10.1038/s41592-025-02795-z PMC12446070 40954301

[80] Tejada-Lapuerta A, Schaar AC, Gutgesell R et al. Nicheformer: a foundation model for single-cell and spatial omics. Nat Methods 2025;22:2525–38. 10.1038/s41592-025-02814-z PMC12695652 41168487

[81] Ouyang J, Wang Y, Gao Y et al. GenAR: next-scale autoregressive generation for spatial gene expression prediction. *arXiv preprint arXiv:2510.04315*, 2025.

[82] Han M, Yang D, Cheng J et al. Towards unified molecule-enhanced pathology image representation learning via integrating spatial transcriptomics. *Pattern Recogn* 2025;172:112458. 10.1016/j.patcog.2025.112458

[83] Weng Z, Fang Y, Qian J et al. HiFusion: hierarchical intra-spot alignment and regional context fusion for spatial gene expression prediction from histopathology. In: Proceedings of the AAAI Conference on Artificial Intelligence (AAAI). Singapore: AAAI Press; 2026, 10630–7.

[84] Huang T, Liu T, Babadi M et al. STPath: a generative foundation model for integrating spatial transcriptomics and whole-slide images. *NPJ Digit Med* 2025;8:659. 10.1038/s41746-025-02020-3 41238784 PMC12618518

[85] Sanian MV, Hemmat A, Vahidi A et al. 3D-guided scalable flow matching for generating volumetric tissue spatial transcriptomics from serial histology. *arXiv preprint arXiv:2511.14613*, 2025.

[86] Liu Y, Wang C, Wang Z et al. High-parameter spatial multi-omics through histology-anchored integration. Nat Methods 2026;23:373–86. 10.1038/s41592-025-02926-6 41407925

[87] Zhu J, Deng R, Guo J et al. SCR-ST: combine single cell with spatial transcriptomics for efficient active sampling via reinforcement learning. *arXiv preprint arXiv:2512.13635*, 2025.

[88] Cheng M, Jiang Y, Jiangshan X et al. Spatially resolved transcriptomics: a comprehensive review of their technological advances, applications, and challenges. *J Genet Genom* 2023;50:625–40. 10.1016/j.jgg.2023.03.011 36990426

[89] Zhao E, Stone MR, Ren X et al. Spatial transcriptomics at subspot resolution with bayesspace. *Nat Biotechnol* 2021;39:1375–84. 10.1038/s41587-021-00935-2 34083791 PMC8763026

[90] Dong K, Zhang S. Deciphering spatial domains from spatially resolved transcriptomics with an adaptive graph attention auto-encoder. *Nat Commun* 2022;13:1739. 10.1038/s41467-022-29439-6 35365632 PMC8976049

[91] Shang L, Zhou X. Spatially aware dimension reduction for spatial transcriptomics. *Nat Commun* 2022;13:7203. 10.1038/s41467-022-34879-1 36418351 PMC9684472

[92] Ji AL, Rubin AJ, Thrane K et al. Multimodal analysis of composition and spatial architecture in human squamous cell carcinoma. *cell* 2020;182:497–514.e22. 10.1016/j.cell.2020.05.039 32579974 PMC7391009

[93] Andersson A, Larsson L, Stenbeck L et al. Spatial deconvolution of HER2-positive breast cancer delineates tumor-associated cell type interactions. Nat Commun 2021;12:6012. 10.1038/s41467-021-26271-2 PMC8516894 34650042

[94] Lake BB, Menon R, Winfree S et al. An atlas of healthy and injured cell states and niches in the human kidney. *Nature* 2023;619:585–94. 10.1038/s41586-023-05769-3 37468583 PMC10356613

[95] Maynard KR, Collado-Torres L, Weber LM et al. Transcriptome-scale spatial gene expression in the human dorsolateral prefrontal cortex. *Nat Neurosci* 2021;24:425–36. 10.1038/s41593-020-00787-0 33558695 PMC8095368

[96] Nagendran M, Sapida J, Arthur J et al. Visium HD enables spatial discovery in FFPE Human Breast Cancer at Single-Cell Scale [EB/OL]. Pleasanton, CA, USA: 10x Genomics; 2023.

[97] Faria M, de Oliveira J, Romero P et al. High-definition spatial transcriptomic profiling of immune cell populations in colorectal cancer. Nat Genet 2025;57:1512–23. 10.1038/s41588-025-02193-3 PMC12165841 40473992

[98] Salas SM, Kuemmerle LB, Mattsson-Langseth C et al. Optimizing xenium in situ data utility by quality assessment and best-practice analysis workflows. *Nat Methods* 2025;22:813–23. 10.1038/s41592-025-02617-2 40082609 PMC11978515

[99] Jaume G, Doucet P, Song A et al. HEST-1k: a dataset for spatial transcriptomics and histology image analysis. *Adv Neural Inf Proces Syst* 2024;37:53798–833.

[100] Chen J, Zhou M, Wenrong W et al. STimage-1K4M: a histopathology image-gene expression dataset for spatial transcriptomics. *Adv Neural Inf Proces Syst* 2024;37:35796–823. 10.52202/079017-1129 PMC12807523 41551654

[101] Andrews TS, Atif J, Liu JC et al. Single-cell, single-nucleus, and spatial RNA sequencing of the human liver identifies cholangiocyte and mesenchymal heterogeneity. *Hepatol Commun* 2022;6:821–40. 10.1002/hep4.1854 34792289 PMC8948611

[102] Janesick A, Shelansky R, Gottscho AD et al. High resolution mapping of the tumor microenvironment using integrated single-cell, spatial and in situ analysis. Nat Commun 2023;14:8353. 10.1038/s41467-023-43458-x PMC10730913 38114474

[103] Shi J, Wei X, Xun Z et al. The web-based portal spatialTME integrates histological images with single-cell and spatial transcriptomics to explore the tumor microenvironment. *Cancer Res* 2024;84:1210–20. 10.1158/0008-5472.CAN-23-2650 38315776

[104] Ke J, Yizhou L, Shen Y et al. ClusterSeg: a crowd cluster pinpointed nucleus segmentation framework with cross-modality datasets. *Med Image Anal* 2023;85:102758. 10.1016/j.media.2023.102758 36731275

[105] Zhu J, Shen Y, Zhang H et al. An anti-biased TBSRTC-category aware nuclei segmentation framework with a multi-label thyroid cytology benchmark. In: International Conference on Medical Image Computing and Computer-Assisted Intervention. Cham: Springer; 2023, 580–90. 10.1007/978-3-031-43987-2_56

[106] Qu C, Zhao R, Yu Y et al. Post-training quantization for 3D medical image segmentation: a practical study on real inference engines. *arXiv preprint arXiv:2501.17343*, 2025.

[107] Deng R, Yang Y. CASC-AI: consensus-aware self-corrective AI agents for noise cell segmentation. *arXiv e-prints*, pages arXiv–2502, 2025.

[108] Xiong J, Deng R, Yue J et al. ZeroReg3D: a zero-shot registration pipeline for 3D consecutive histopathology image reconstruction. *J Med Imaging* 2025;12:044002–2. 10.1117/1.JMI.12.4.044002 PMC12322837 40765693

[109] Wang C, Cui H, Zhang A et al. scGPT-spatial: continual pretraining of single-cell foundation model for spatial transcriptomics. *biorxiv* 2025;2025–02.

[110] Madhu H, Rocha JF, Huang T et al. HEIST: a graph foundation model for spatial transcriptomics and proteomics data. *ArXiv*, pages arXiv–2506, 2025.

[111] Zhao S, Luo, Y, Yang G et al. SToFM: a multi-scale foundation model for spatial transcriptomics. *arXiv preprint arXiv:2507.11588*, 2025.

[112] Zhang N, Long Y, Xia S et al. Inferring spatial gene expression from tissue images using large-scale histology foundation model with spafoundation. *bioRxiv* 2025;2025–08.

[113] Wang Y, Wang J, Yanyu X et al. FmH2ST: foundation model-based spatial transcriptomics generation from histological images. Nucleic Acids Res 2025;53:gkaf865. 40923764 10.1093/nar/gkaf865 PMC12418390

[114] Liu T, Huang T, Ding T et al. Leveraging multi-modal foundation models for analysing spatial multi-omic and histopathology data. *Nat Biomed Eng* 2026;1–18. 41644824 10.1038/s41551-025-01602-6

[115] Hemker K, Song AH, Almagro-Pérez C et al. Towards spatial transcriptomics-driven pathology foundation models. *arXiv preprint arXiv:2602.14177*, 2026.

[116] Li Z, Wu W, Cui Y et al. SOFisher: reinforcement learning-guided experiment designs for spatial omics. *bioRxiv* 2024;2024–07. 10.1038/s41467-026-73404-6 PMC13388978 42185268

[117] Yuan M, Jin K, Yan H et al. Smart spatial omics (S2-omics) optimizes region of interest selection to capture molecular heterogeneity in diverse tissues. *Nat Cell Biol* 2025;27:2225–38. 10.1038/s41556-025-01811-w 41298871 PMC12662399

[118] Huang CH . QuST-LLM: integrating large language models for comprehensive spatial transcriptomics analysis. *arXiv preprint arXiv:2406.14307*, 2024.

[119] Xiao Y, Liu J, Zheng Y et al. CellAgent: an LLM-driven multi-agent framework for automated single-cell data analysis. *arXiv preprint arXiv:2407.09811*, 2024.

[120] Lin Z, Wang W, Marin-Llobet A et al. Spatial transcriptomics AI agent charts HPSC-pancreas maturation in vivo. *bioRxiv* 2025;2025–04.

[121] Yang C, Zhang X, Chen J. ChatSpatial: schema-enforced agentic orchestration for reproducible and cross-platform spatial transcriptomics. *bioRxiv* 2026;2026–02.

[122] Zhang D, Zhang M, Li N et al. EnsAgent: a tool-ensemble multiple agent system for robust annotation in spatial transcriptomics. *bioRxiv* 2026;2026–03.

[123] Jordan J, Smith XW, McPheeters M et al. stMCP: spatial transcriptomics with a model context protocol server. *bioRxiv* 2026;2026–03.

[124] Song AH, Jaume G, Williamson DFK et al. Artificial intelligence for digital and computational pathology. *Nat Rev Bioeng* 2023;1:930–49.

[125] Kanemaru K, Cranley J, Muraro D et al. Spatially resolved multiomics of human cardiac niches. *Nature* 2023;619:801–10. 10.1038/s41586-023-06311-1 37438528 PMC10371870

[126] Heiser CN, Simmons AJ, Revetta F et al. Molecular cartography uncovers evolutionary and microenvironmental dynamics in sporadic colorectal tumors. *Cell* 2023;186:5620–5637.e16. 10.1016/j.cell.2023.11.006 38065082 PMC10756562

[127] Gao H, Liu Z, Van Der Maaten L et al. Densely connected convolutional networks. In: Proceedings of the IEEE conference on computer vision and pattern recognition (CVPR). Honolulu, HI, USA: IEEE; 2017, 4700–8.

[128] Jia Y, Liu J, Chen L et al. THItoGene: a deep learning method for predicting spatial transcriptomics from histological images. Brief Bioinform 2023;25:bbad464. 10.1093/bib/bbad464 PMC10749789 38145948

[129] Radford A, Kim JW, Hallacy C et al. Learning transferable visual models from natural language supervision. In: *International Conference on Machine Learning (ICML)*. Virtual Event: PMLR; 2021, 8748–63.

[130] Wen H, Tang W, Jin W et al. Single cells are spatial tokens: transformers for spatial transcriptomic data imputation. *arXiv preprint arXiv:2302.03038*, 2023.

[131] Ho J, Jain A, Abbeel P. Denoising diffusion probabilistic models. *Adv Neural Inf Proces Syst* 2020;33:6840–51.

[132] Peebles W, Xie S. Scalable diffusion models with transformers. In: Proceedings of the IEEE/CVF International Conference on Computer Vision, (ICCV). Paris, France: IEEE/CVF; 2023, 4195–205.

[133] Lipman Y, Chen RTQ, Ben-Hamu H et al. Flow matching for generative modeling. *arXiv preprint arXiv:2210.02747*, 2022.

[134] Wang C, Chan AS, Xiaohang F et al. Benchmarking the translational potential of spatial gene expression prediction from histology. Nat Commun 2025;16:1544. 10.1038/s41467-025-56618-y PMC11814321 39934114

[135] Xue S, Zhu F, Chen J et al. Inferring single-cell resolution spatial gene expression via fusing spot-based spatial transcriptomics, location, and histology using GCN. Brief Bioinform 2024;26:bbae630. 10.1093/bib/bbae630 PMC11645551 39656774

[136] Zeira R, Land M, Strzalkowski A et al. Alignment and integration of spatial transcriptomics data. *Nat Methods* 2022;19:567–75. 10.1038/s41592-022-01459-6 35577957 PMC9334025

[137] Vaswani A, Shazeer N, Parmar N et al. Attention is all you need.Adv Neural Inf Process Syst 2017;30:6000–10.

[138] Zhao Y, Alizadeh E, Liu Y et al. Inferring single-cell spatial gene expression with tissue morphology via explainable deep learning. *BioRxiv* 2024;2024–06.

[139] Noh M, Lee S, Kim S et al. PathCLAST: pathway-augmented contrastive learning with attention for interpretable spatial transcriptomics. *Brief Bioinform* 2026;27:bbag029. 41627343 10.1093/bib/bbag029 PMC12862980

[140] Jain S, Wallace BC. Attention is not explanation. In: Proceedings of the 2019 Conference of the North American Chapter of the Association for Computational Linguistics: Human Language Technologies, Volume 1 (Long and Short Papers) (NAACL-HLT). Minneapolis, MN, USA: Association for Computational Linguistics; 2019, 3543–56.

[141] Ramprasaath R, Selvaraju MC, Das A et al. Grad-CAM: visual explanations from deep networks via gradient-based localization. In: Proceedings of the IEEE International Conference on Computer Vision (ICCV). Venice, Italy: IEEE; 2017, 618–26.

[142] Sundararajan M, Taly A, Yan Q. Axiomatic attribution for deep networks. In: *International Conference on Machine Learning (ICML)*. Sydney, Australia: PMLR; 2017, 3319–28.

[143] Hallinan C, Lucas C-HG, Fan J. Impact of Data Quality on Deep Learning Prediction of Spatial Transcriptomics from Histology Images. bioRxiv, 2025.

[144] Schmauch B, Romagnoni A, Pronier E et al. A deep learning model to predict RNA-seq expression of tumours from whole slide images. *Nat Commun* 2020;11:3877. 10.1038/s41467-020-17678-4 32747659 PMC7400514

[145] Ribeiro MT, Singh S, Guestrin C. ”Why should I trust you?” explaining the predictions of any classifier. In: Proceedings of the 22nd ACM SIGKDD International Conference on Knowledge Discovery and Data Mining, (KDD). San Francisco, CA, USA: ACM; 2016, 1135–44.

[146] Lundberg SM, Lee S-I. A unified approach to interpreting model predictions. Adv Neural Inf Process Syst 2017;30:4768–77.

[147] Tan X, Mulay O, Xie J et al. Robust and interpretable prediction of gene markers and cell types from spatial transcriptomics data. Nat Commun 2026;17:1781. 10.1038/s41467-026-68487-0 PMC12917206 41545411

[148] Duan B, Cheng X, Zhou H. SpaPheno: linking spatial transcriptomics to clinical phenotypes with interpretable machine learning. *bioRxiv* 2025;2025–09. 10.1186/s13073-026-01645-7 PMC13185361 41975540

[149] Patkar S, Chen A, Basnet A et al. Predicting the tumor microenvironment composition and immunotherapy response in non-small cell lung cancer from digital histopathology images. NPJ Precis Oncol 2024;8:280. 10.1038/s41698-024-00765-w PMC11659524 39702609

[150] Li L, Dong L, Zhang H et al. spaLLM: enhancing spatial domain analysis in multi-omics data through large language model integration. Brief Bioinform 2025;26:bbaf304. 40608008 10.1093/bib/bbaf304 PMC12224616

[151] Li L, Wang T, Liang Z et al. FineST: contrastive learning integrates histology and spatial transcriptomics for nuclei-resolved ligand-receptor analysis. *Nat Commun* 2026. 10.1038/s41467-026-70528-7 PMC13201544 41839892

[152] Guo B, Ling W, Kwon SH et al. Integrating spatially-resolved transcriptomics data across tissues and individuals: challenges and opportunities. *ArXiv*, pages arXiv–2408, 2024.

[153] Guo T, Yuan Z, Pan Y et al. Spiral: integrating and aligning spatially resolved transcriptomics data across different experiments, conditions, and technologies. Genome Biol 2023;24:241. 37864231 10.1186/s13059-023-03078-6 PMC10590036

[154] Ma H, Zhang X, Yilong Q et al. Vispro improves imaging analysis for visium spatial transcriptomics. Genome Biol 2025;26:173. 10.1186/s13059-025-03648-w PMC12177973 40533768

[155] Bhuva DD, Tan CW, Salim A et al. Library size confounds biology in spatial transcriptomics data. Genome Biol 2024;25:99. 10.1186/s13059-024-03241-7 PMC11025268 38637899

[156] Millard N, Chen JH, Palshikar MG et al. Batch correcting single-cell spatial transcriptomics count data with crescendo improves visualization and detection of spatial gene patterns. Genome Biol 2025;26:36. 10.1186/s13059-025-03479-9 PMC11863647 40001084

[157] Yunfei H, Xie M, Li Y et al. Benchmarking clustering, alignment, and integration methods for spatial transcriptomics. Genome Biol 2024;25:212. 10.1186/s13059-024-03361-0 PMC11312151 39123269

[158] Smith KD, Prince DK, MacDonald JW et al. Challenges and opportunities for the clinical translation of spatial transcriptomics technologies. *Glomerular Dis* 2024;4:49–63. 10.1159/000538344 38600956 PMC11006413

[159] Isnard P, Humphreys BD. Spatial transcriptomics: integrating morphology and molecular mechanisms of kidney diseases. *Am J Pathol* 2025;195:23–39. 10.1016/j.ajpath.2024.06.012 39097166 PMC12179522

[160] Campanella G, Chen S, Singh M et al. A clinical benchmark of public self-supervised pathology foundation models. *Nat Commun* 2025;16:3640. 10.1038/s41467-025-58796-1 40240324 PMC12003829

[161] Li Z, Li Y, Xiang J et al. AI-enabled virtual spatial proteomics from histopathology for interpretable biomarker discovery in lung cancer. *Nat Med* 2026;32:231–44. 10.1038/s41591-025-04060-4 41491099 PMC12823406

[162] Andani S, Chen B, Ficek-Pascual J et al. Histopathology-based protein multiplex generation using deep learning. *Nat Mach Intell* 2025;7:1292–307. 40842484 10.1038/s42256-025-01074-y PMC12364712

[163] Eric W, Bieniosek M, Zhenqin W et al. ROSIE: AI generation of multiplex immunofluorescence staining from histopathology images. Nat Commun 2025;16:7633. 40819165 10.1038/s41467-025-62346-0 PMC12357954

[164] Valanarasu JMJ, Hanwen X, Usuyama N et al. Multimodal AI generates virtual population for tumor microenvironment modeling. *Cell* 2026;189:386–400.e19. 10.1016/j.cell.2025.11.016 41371214

[165] Jaume G, Oldenburg L, Vaidya A et al. Transcriptomics-guided slide representation learning in computational pathology. In: Proceedings of the IEEE/CVF Conference on Computer Vision and Pattern Recognition (CVPR). Seattle, WA, USA: IEEE/CVF; 2024, 9632–44.

[166] Pan S, Chen J, Secrier M. Teaching pathology foundation models to accurately predict gene expression with parameter efficient knowledge transfer. In: International Conference on Medical Image Computing and Computer-Assisted Intervention. Cham: Springer; 2025, 605–13. 10.1007/978-3-032-04981-0_57

